# Human trafficking for labour exploitation: the results of a two-phase systematic review mapping the European evidence base and synthesising key scientific research evidence

**DOI:** 10.1007/s11292-017-9321-3

**Published:** 2018-04-06

**Authors:** Ella Cockbain, Kate Bowers, Galina Dimitrova

**Affiliations:** Department of Security and Crime Science, University College London (UCL), 35 Tavistock Square, London, WC1H 9EZ UK

**Keywords:** Exploitation, Forced labour, Human trafficking, Immigration, Migration, Organised crime, Modern slavery, Servitude, Systematic review, Systematic map

## Abstract

**Objectives:**

Our objectives were (1) to systematically map the contours of the European evidence base on labour trafficking, identifying its key characteristics, coverage, gaps, strengths and weaknesses and (2) to synthesise key scientific research.

**Methods:**

We took a two-phase approach: a systematic map followed by a detailed synthesis of key scientific research evidence. Our search strategy included 15 databases, hand searches of additional journals, backwards searches, snowball searches and expert recommendations. We identified and screened 6106 records, mapped 152 and synthesised eight.

**Results:**

Overall, the literature was limited and fragmented. Reports produced by official agencies dominated; academic authorship and peer-reviewed outputs were comparatively rare. Few publications met minimum scientific standards. Qualitative designs outweighed quantitative ones. Publications typically described trafficking’s problem profile and/or discussed interventions; they rarely assessed trafficking’s impacts or evaluated interventions. Even among the key scientific research, the quality of evidence was variable and often low. Particular weaknesses included poor methods reporting, unclear or imprecise results and conclusions not properly grounded in the data. The synthesised studies were all exploratory, also sharing other design features. Common themes identified included: poor treatment of victims; diversity of sectors affected and commonalities among victims; inadequacies of current responses; and barriers to interventions.

**Conclusions:**

There is a lack of high-quality studies into European labour trafficking. Methodological opacity, insufficient rigour and publication in non-indexed locations impede the identification, assessment and synthesis of evidence. Adherence to higher reporting standards would further the field’s development and particular research gaps should be addressed.

**Electronic supplementary material:**

The online version of this article (10.1007/s11292-017-9321-3) contains supplementary material, which is available to authorized users.

## Introduction and background

Human trafficking, sometimes known as modern slavery^1^, is often framed as one of the greatest organised crime threats facing modern society. Numerous reports and, to a lesser extent, research studies have argued that human trafficking adversely affects the stability, security and well-being of individual victims, entire communities, industries and even nations (Belser 2005; Home Office 2012; Kelly 2002; Zimmerman et al. 2006). With increases in political, media and public attention since the 1990s has come a host of legislation and policy-making designed to tackle trafficking (Goodey 2008; Van Der Laan et al. 2011). Yet, the demand for robust research to inform evidence-based policy and practice far outpaces its supply. Debate and decision-making often rely instead on anecdotal evidence, supposition, rhetoric or popular wisdom.

Systematic reviews of any aspect of the trafficking literature are rare; notable exceptions have focused on trafficking’s health impacts (Oram et al. 2012a; Ottisova et al. 2016), interventions around sex trafficking (Macy and Graham 2012; Van Der Laan et al. 2011) and the state of trafficking research more broadly (Gozdziak and Bump 2008). In contrast, non-systematic reviews of the trafficking literature and commentaries are fairly common but have greater potential for bias in the identification, selection, assessment and interpretation of evidence (Gough et al. 2012). These literature reviews’ key findings proved helpful in informing our systematic review’s design and providing context.

Among the most common criticisms of the trafficking literature are the limited focus and coverage (e.g. thematic and geographical skews), the predominance of purely descriptive work, methodological opacity, weak research design (e.g. questionable assumptions, inappropriate datasets), sampling biases (a common shortcoming when working with hidden populations), ill-founded inferences, a tendency to be emotionally and/or politically charged and lack balance, and poor-quality statistics (e.g. projections, estimates with huge ranges, lack of rigour in their generation and interpretation) (Andrees and van der Linden 2005; Aronowitz 2001, 2009; Di Nicola 2007; Feingold 2010; Goodey 2008; Kelly 2005; Laczko and Gozdziak 2005; Lehti and Aromaa 2006; Tyldum and Brunovskis 2005).

The overwhelming focus on sex trafficking has left other trafficking types overlooked (Andrees and Linden 2005; Home Office 2007; Kelly 2005; Laczko and Gozdziak 2005; Surtees 2008). Major knowledge gaps persist around even basic aspects of labour trafficking, for example, let alone the effectiveness of counter-measures (Andrees and Linden 2005; Home Office 2007; Kelly 2005; Laczko and Gozdziak 2005). A non-systematic review of the United Kingdom’s (UK) literature on labour trafficking identified just nine studies, none of which included quantitative analysis (Home Office 2007).

Yet, labour trafficking is increasingly prioritised at national and international levels (European Commission 2012; Home Office 2011a, 2011b, 2014; U.S. Department of State 2014). In the UK, for example, 2014’s overall rise in trafficking referrals included a particularly steep growth in suspected labour trafficking cases (National Crime Agency 2015). Analysing the characteristics of the 2727 trafficking victims^2^ identified in the UK from 2009 to 2014, Cockbain and Bowers (2015) found that the most common exploitation type overall was labour (44%), ahead of both sex (41%) and domestic servitude (12%). Several other European Union member states have reported that labour trafficking is on the rise (European Commission 2016). Countries and regions appear to vary greatly, however, both in the volume of identified labour trafficking cases and what proportion of the local trafficking problem they represent (Belser et al. 2005; de Jonge 2005; United Nations Office on Drugs and Crime 2012, 2014).

We identified a clear need for a systematic review of the evidence on labour trafficking. We decided to focus on Europe for several reasons. First, we were aware of a particular demand for such a product here as labour trafficking had been singled out as a priority in the European Union’s counter-trafficking strategy (European Commission 2012) and in EUROPOL’s operational action plan on trafficking (Government of the Netherlands 2016). Second, trafficking has pronounced regional variation and, given the European interest, it made sense to focus on European evidence. Third, a narrower geographical remit was a useful, pragmatic counterbalance to our review’s otherwise broad thematic scope and the diverse array of evidence we intended to consider.

Our overarching review objectives were to assess the shape of the overall evidence base on European labour trafficking, identifying coverage and gaps, strengths and weaknesses and to synthesise key scientific research evidence on the problem and associated counter-measures. We favoured an exploratory two-phase approach (a systematic map followed by a targeted synthesis), as recommended for reviews like ours that deal with broad topics and poorly-charted domains (EPPI-Centre 2010; Gough and Thomas 2012; Oliver and Sutcliffe 2012). The particular contributions of the mapping stage are outlining the contours of the literature and informing the direction and interpretation of the synthesis (Gough 2007; Gough and Thomas 2012; Oliver and Sutcliffe 2012).

Our review questions were:

1. What is the overall state of the empirical evidence base on the scale, nature and impacts of labour trafficking affecting Europe and on-the-ground counter-measures? (Systematic map)
2. What can we learn from key scientific research evidence about the scale, nature and impacts of labour trafficking affecting Europe and on-the-ground counter-measures? (Synthesis)

We defined ‘human trafficking’ in accordance with international law as:

the recruitment, transportation, transfer, harbouring or receipt of persons, by means of the threat or use of force or other forms of coercion, of abduction, of fraud, of deception, of the abuse of power or of a position of vulnerability or of the giving or receiving of payments or benefits to achieve the consent of a person having control over another person, for the purpose of exploitation. Exploitation shall include, at a minimum, the exploitation of the prostitution of others or other forms of sexual exploitation, forced labour or services, slavery or practices similar to slavery, servitude or the removal of organs. (United Nations 2000, p. 42)

There is no equivalent agreed and specific definition of ‘labour trafficking’. Even within the European Union, there is variation between and within states in what is seen to constitute trafficking for labour exploitation. Particular discrepancies exist around whether the term covers exploitation in criminal industries (e.g. pickpocketing, cannabis cultivation) and/or within the household (domestic servitude). Our review definition of labour trafficking was that implicitly used in the UK for data collection and case monitoring (via the ‘National Referral Mechanism’): exploitation of any bodily labour except sexual services and domestic servitude. This definition covers labour in both legal and illegal industries but excludes domestic servitude. Analysis of UK trafficking data has revealed statistically significant differences between victims of domestic servitude and labour trafficking (Cockbain and Bowers 2015). Such results suggest that treating domestic servitude as part of labour trafficking risks conflating two potentially divergent issues and obscuring key differences. It is also worth noting that, although there is conceptual overlap between ‘labour trafficking’ and ‘forced or compulsory labour’ (as defined in international law^3^), the two issues are not equivalent and each can occur without the other.

We defined ‘Europe’ as all European Union (EU) member states, candidates and potential candidates plus additional countries not affiliated with the EU but located within the geographical territory commonly understood as Europe (e.g. Switzerland, Norway, Russia). The full list of countries covered is:

Albania, Andorra, Austria, Belarus, Belgium, Bosnia and Herzegovina, Bulgaria, Croatia, Cyprus, Czech Republic, Denmark, Estonia, Finland, France, Germany, Greece, Holy See, Hungary, Iceland, Ireland, Italy, Kosovo, Latvia, Liechtenstein, Lithuania, Luxembourg, Macedonia (The Former Yugoslav Republic of), Malta, Moldova, Monaco, Montenegro, Netherlands, Norway, Poland, Portugal, Romania, Russian Federation, San Marino, Serbia, Slovakia, Slovenia, Spain, Sweden, Switzerland, Turkey, Ukraine and the UK.

## Methods

### Ethics, review registration and protocol

Ethical approval was not required for this project. We registered our review prospectively in the PROSPERO International Prospective Register of Systematic Reviews (Cockbain et al. 2014), although our eventual review was far more comprehensive than what we had initially planned and registered. Our protocol adhered to the Preferred Reporting Items for Systematic Review and Meta-analysis Protocols (PRISMA-P) guidelines (see Appendix 1 for the checklist). There is no equivalent PRISMA tool for systematic mapping.

### Stakeholder engagement

We engaged with key stakeholders (over 100 in total) to ensure external scrutiny, draw on their diverse perspectives and experiences and maximise the review’s relevance and practical application (Rees and Oliver 2012). Table 1 contains details about the stakeholder groups and their composition. We initially consulted with the EMPACT group and the UK Threat Group on our review aims and design. Later, both these two groups and the additional experts had the opportunity to review our preliminary findings and plans for targeted synthesis, provide feedback and identify any outstanding publications for consideration.

**Table 1:** Stakeholder engagement

Group of stakeholders	Description of group	Number of members consulted	Countries represented
EMPACT Initiative	Formal group of counter-trafficking leads tasked with coordinating and improving European responses to human trafficking. Vast majority of members from national law enforcement but group also includes representatives of transnational organisations like EUROPOL, CEPOL and EUROJUST.	71	Belgium, Bulgaria, Cyprus, Czech Republic, Denmark, France, Ireland, Hungary, Latvia, Luxembourg, Malta, Netherlands, Poland, Portugal, Romania, Slovak Republic, Slovenia, Spain, Sweden, Switzerland, UK.
UK Threat Group on Human Trafficking	Formal multi-agency group that leads the UK’s strategic response to human trafficking. Members include key representatives from the Home Office, Foreign Office, Gangmasters’ Licencing Authority, tax authorities, police, National Crime Agency, Crown Prosecution Service and the Child Trafficking Advice Centre.	30	UK.
Additional experts	Not a formal group but rather a set of academics and other researchers with clear expertise on labour trafficking. Identified on an ad hoc basis through their publications and/or discussions at various trafficking related conferences and events.	6	Austria, Finland, Netherlands, UK.

### Search strategy

We used varied and complementary search strategies, designed to retrieve relevant publications from both the academic and grey literatures. First, we ran keyword searches of the following fifteen databases:

1. ASSIA (Applied Social Sciences Index and Abstracts)
2. Continental Europe Database
3. Criminal Justice Database
4. East Europe, Central Europe Database
5. ERIC (Education Resources Information Center)
6. IBSS (International Bibliography of the Social Sciences)
7. NCJRS (National Criminal Justice Reference System) Abstracts Database
8. Proquest theses and dissertations
9. PsycINFO
10. PsycEXTRA
11. SCOPUS
12. Social Policy and Practice
13. Social Science Database
14. Sociological Abstracts
15. Web of Science.

Second, we conducted manual searches of six journals, a selection chosen because we knew them to contain trafficking research but they were not (fully) indexed in the above databases. They were:* Brown Journal of World Affairs*;* Criminology and Public Policy*;* Policing*:* A Journal of Policy and Practice*;* Health and Human Rights*;* International Health*; and* Journal of Immigrant and Minority Health*. Third, we ran backwards searches on the contents of two major bibliographies we found that catalogued publications on human trafficking (Farquet et al. 2005; Gozdziak et al. 2015). Fourth, we conducted backwards searches on any trafficking-related reviews we encountered and snowball searches of the other publications we mapped. Finally, we asked stakeholders if they knew of any further relevant publications we had missed.

The range of issues that labour trafficking encompasses and the lack of definitional consistency and clarity meant we needed broad and varied search terms. Our search terms (Appendix 2) were designed to capture synonyms, variants and closely associated issues around both of the two fundamental constructs involved in labour trafficking, namely human trafficking and labour exploitation. We were aware that publications might deal, for example, with ‘trafficking in agriculture’ without specifically (and arguably redundantly) labelling this phenomenon ‘labour trafficking’. As a safeguard, we designed our search terms to also include, as an alternative to the generic category ‘labour’, some specific industries commonly associated with labour trafficking in the academic and grey literature (Andrees and Linden 2005; Home Office 2007, 2012; National Crime Agency 2014), policy discourse and media debate.

### Inclusion criteria and study selection

We uploaded search results to specialist systematic review software (EPPI 4 Reviewer). Most of our results came from database searches and were returned in a format suited to screening on title and abstract; this worked as an initial sift before full text screening. Results from other sources typically lacked abstracts and so we proceeded directly to full text screening. We used the sequence of inclusion criteria detailed in Table 2. The first criterion was built into the searches and the second was applicable to full text screening only, whereas all the rest were used for both title and abstract and full text screening.

**Table 2:** Inclusion criteria for the systematic map

Inclusion criterion	Summary	Further details/explanation
1. Publication date	Publication between 1 January 2000 and 13 July 2015	End date is when our searches began. Start date is a key year for trafficking: international and legally-binding consensus on what constitutes trafficking was finally reached in 2000 (United Nations 2000). Until then, definitions were so notoriously divergent (Aronowitz 2001) that including earlier publications could have undermined the comparability of the studies reviewed.
2. Accessibility	Full text accessible	We conducted extensive searches via the British Library, our institutional library/e-library, commercial booksellers, specific relevant websites and general search engines. If we could not find a full text in this way, we contacted authors directly requesting a copy. Only if all this did not work did we exclude a text for being inaccessible.
3. Broad relevance	Addresses modern day human trafficking and uses data generated from 1990 onwards	We chose 1990 to give a reasonable window for data collection for studies from the start of our publication date range while still retaining a focus on modern day trafficking and filtering out material on earlier forms of unfree labour (e.g. slavery in classical Greece or nineteenth century America) that might feasibly meet the research definition (United Nations 2000) but would not be considered human trafficking in the standard sense.
4. Specificity	Contains material specifically about trafficking for labour exploitation, rather than for other ends (e.g. sex) or just in general terms.	Our research definition of labour excluded domestic servitude, but there is little consensus about the parameters of what constitutes labour trafficking. To maintain focus but support an inclusive approach to evidence, we excluded publications dealing *exclusively* with trafficking for domestic servitude but included those that addressed labour trafficking in a way that also covered, implicitly or explicitly, domestic servitude.
5. Geography	Deals with labour trafficking into, within or from a European country.	One or more countries that we defined as European countries features in the publication as a site for primary data collection or source of secondary data.
6. Language	Is in English	For practical reasons we only included English language publications. This language restriction introduces constraints. To have conducted a fully inclusive review on this front, we would have needed the capacity to run searches in (at a minimum) all European languages, screen results and process the contents of those qualifying for inclusion. Considering the number of languages spoken across Europe, this would have been an untenable undertaking.
7. Empirical data	Must contain empirical data on European labour trafficking	Under the category ‘empirical data’ we included evaluations, reviews (systematic or not) and other primary research, as well as empirically-grounded descriptions of administrative and other data. We excluded entirely theoretical pieces, commentaries, training manuals and handbooks, news reports, media content analyses and work concerned with anti-trafficking law or policy in purely normative terms.
8. No double counting	Is the single most relevant and empirically rich publication from a given study	If we found multiple publications that used data from the same enquiry, we selected for inclusion the one that we deemed the most relevant and empirically rich. In some cases, we came across multi-country studies that resulted in the publication of single-country studies *and* multi-country overviews/comparative analyses. In such instances, we used the multi-country overviews.

Each result was screened by one of two reviewers. We first piloted our screening codebook^4^ by double-screening 50 studies. We identified, discussed and resolved sources of disagreement and refined the codebook as necessary. We later tested for inter-rater reliability by double-coding a randomly selected 5% (*n* = 216) of the total records screened on title and abstract. The results indicated strong consistency between coders (McHugh 2012): 95% agreement on inclusion/exclusion and a Cohen’s kappa value of 0.8.

### An overview of our review process

**Fig. 1:**
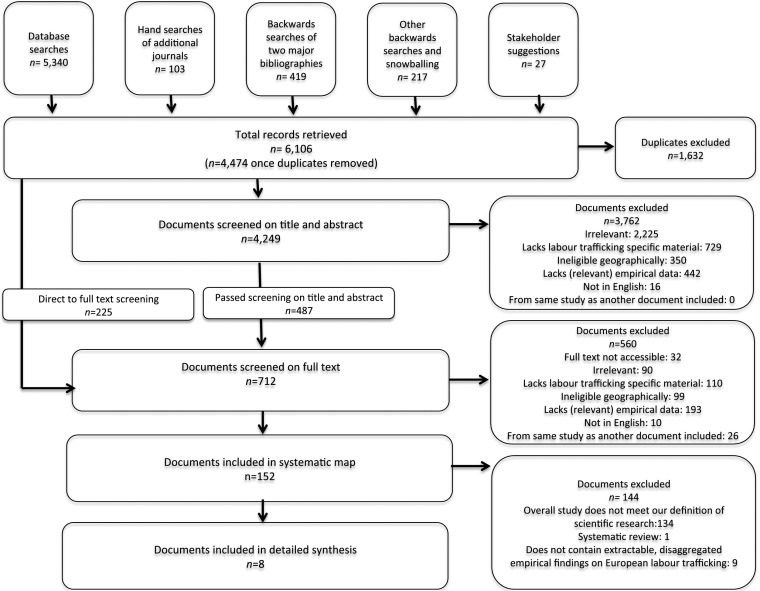
Flow of documents through our review

Figure 1 shows the flow of documents through the review. Our searches yielded 6106 records; once we had removed duplicates, 4474 unique publications remained. We mapped 152 of them and synthesised eight.

#### Phase 1: the systematic map

We mapped the full texts using a detailed data extraction form (Appendix 3). A single reviewer coded the text for the map. For quality assurance purposes, a second reviewer double-coded a randomly selected 10% (*n* = 15) of texts. A comparison of the exact codes assigned showed a high degree of consistency between coders (86%).

#### Phase 2: the detailed synthesis

**Fig. 2:**
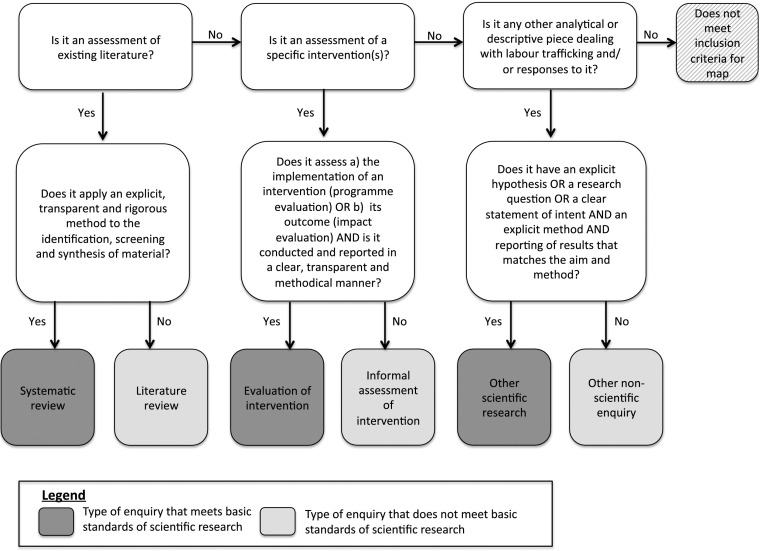
Categorisation of different types of enquiry

The systematic map highlighted the methodological weaknesses of the literature, which influenced how we targeted our synthesis. We selected studies for inclusion using the criteria in Table 3. Figure 2 is a graphical depiction of how we assessed publications against the first criterion in Table 3: whether or not they contained ‘scientific research’.

**Table 3:** Inclusion criteria for the targeted synthesis

Inclusion criterion	Summary	Further details/explanation
1. Research meets basic scientific standards	Must contain ‘scientific research’	This category comprised systematic reviews, evaluations of interventions and other scientific research. All qualifying publications met basic scientific standards in terms of methods and reporting and as such provided a relatively strong and reliable basis for synthesis. See Fig. 2 for further explanation of how the different types of enquiry were assessed.
2. No double counting	Must not be a systematic review	To avoid double counting of evidence that might skew the results, it is common practice to exclude other systematic reviews from synthesis and use instead any of the original studies that qualify for inclusion.
3. Focused results around European labour trafficking	Must contain disaggregated, extractable, substantive empirical evidence on European labour trafficking	In order to be of value to our synthesis, it was vital that we could extract from the publications empirical evidence specific to our research focus (European labour trafficking) rather than aggregate data combining this issue with other forms of trafficking and/or other geographies. We also used the requirement of substantiveness to decide whether a publication had sufficient relevant results to enrich the synthesis, rather than cluttering it for little added value. Due to the subjectivity around how much is enough to be substantiveness, any texts considered for exclusion on this basis were discussed by the review team. An illustrative example of a publication excluded on this basis was Gjermeni et al. (2008, p. 945), in which the sum total of results specific to European labour trafficking was the sentences: ‘In terms of type of work, young children (ages 6–11) were engaged in begging while older youth were involved in theft, trade in drugs, street vending and prostitution. Boys were especially likely to be engaged in selling on the streets and begging’.

Eight publications met the inclusion criteria for synthesis. They varied in their design, methods and foci and included substantial qualitative material. Consequently, we designed our data extraction form (Appendix 4) to be broad and inclusive, simple and to allow for narrative detail. Our intention was to draw out further methodological information and summarise key findings, conclusions and recommendations. Where studies included but were not limited to European labour trafficking, we coded in relation to material on this topic only. Two studies in the sample were book chapters (overviews of multi-country studies) (Jokinen and Ollus 2011; Rijken 2011); in both cases, we used the entire books to get methodological information and complete the quality assessment. One reviewer did the coding in full and a second reviewer revised and commented on their outputs for validation and quality assurance. The few discrepancies that arose relating to what the two reviewers saw as key results and conclusions were easily resolved in discussion.

Given the anticipated nature of the literature, we chose to assess the quality of the studies based on the best methodological approach possible under the circumstances of their designs, rather than the risk of bias inherent in these designs in the first place (for further discussion of the distinction between the two approaches, please see Liberati et al. 2009). Our quality assessment tool (Appendix 5) was based loosely on that from a systematic review of the health impacts of trafficking (Oram et al. 2012a). By rephrasing certain criteria and removing others, we simplified the original tool and made it applicable to a broader range of topics and methods. For example, we deleted the criterion ‘Are the findings generalisable?’ since generalisability is not something against which qualitative research is usually or reasonably assessed.

Two coders independently quality assessed each study. The results indicated a high level of inter-rater consistency: the rank order of the studies (based on total score) was the same and there was 88% overall agreement between coders. All discrepancies, none of which were larger than one point, were discussed and resolved.

## Results

We present results from the systematic map and synthesis in turn. We use examples from publications to substantiate certain findings: these were selected for context and illustrative purposes and are neither exhaustive nor intended to ‘name and shame’. Our coding was necessarily based on what was *reported* in the publications. It remains a possibility that at least some of the publications with underdeveloped or missing methods section were less methodologically weak in practice than in their reporting.

### Phase 1: Systematic map

The five key findings presented here together describe the overall state of the empirical evidence base on labour trafficking affecting Europe.

**Fig. 3:**
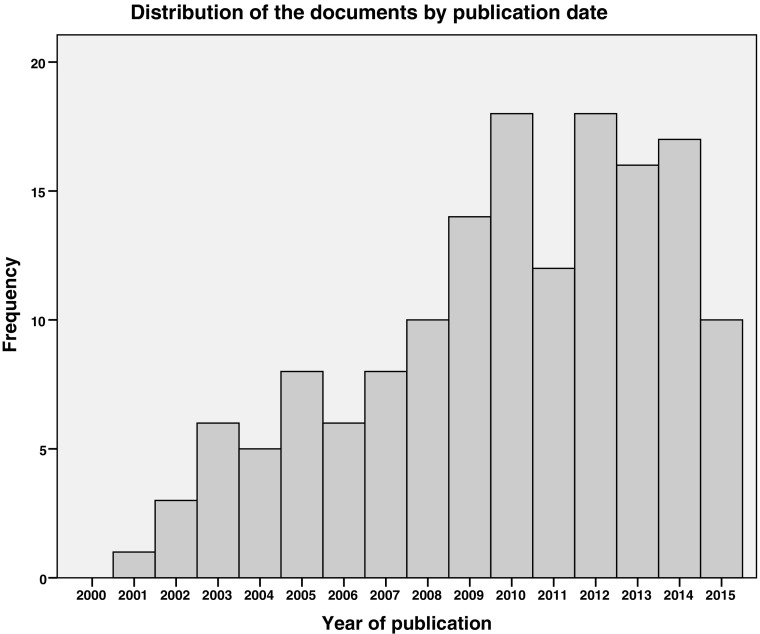
Distribution of the documents by publication date
  The publications used data collected in and/or from a wide range of European countries, including both developed and transition states (see Fig. 4)^5^. The geographical distribution of coverage within Europe was fairly even, with a median of 23 publications per country and an interquartile range of 7 (20–27). There were notable outliers at both ends: the UK featured 78 times and five countries (Andorra, Holy See, Lichtenstein, Monaco, San Marino) appeared only once or not at all.

1. Although the empirical evidence base on labour trafficking is underdeveloped, the issue has been addressed in the context of numerous and diverse European countries
  Despite the breadth of our map question, search strategies and inclusion criteria, we identified just 152 publications containing relevant empirical evidence on European labour trafficking (full bibliography in Appendix 6). There was no strong temporal trend in the production of evidence (see Fig. 3) but a slight skew towards later years: almost 70% (*n* = 105) of publications appeared from January 2009 to July 2015 (approximately 40% of the study period). During screening, we noted that certain countries’ annual reports of human trafficking originally dealt exclusively with sex trafficking: in Sweden and the Netherlands, for example, labour trafficking was first included respectively in the ninth (National Criminal Police 2007) and fifth (Dettmeijer-Vermeulen et al. 2006) such reports.

**Fig. 4:**
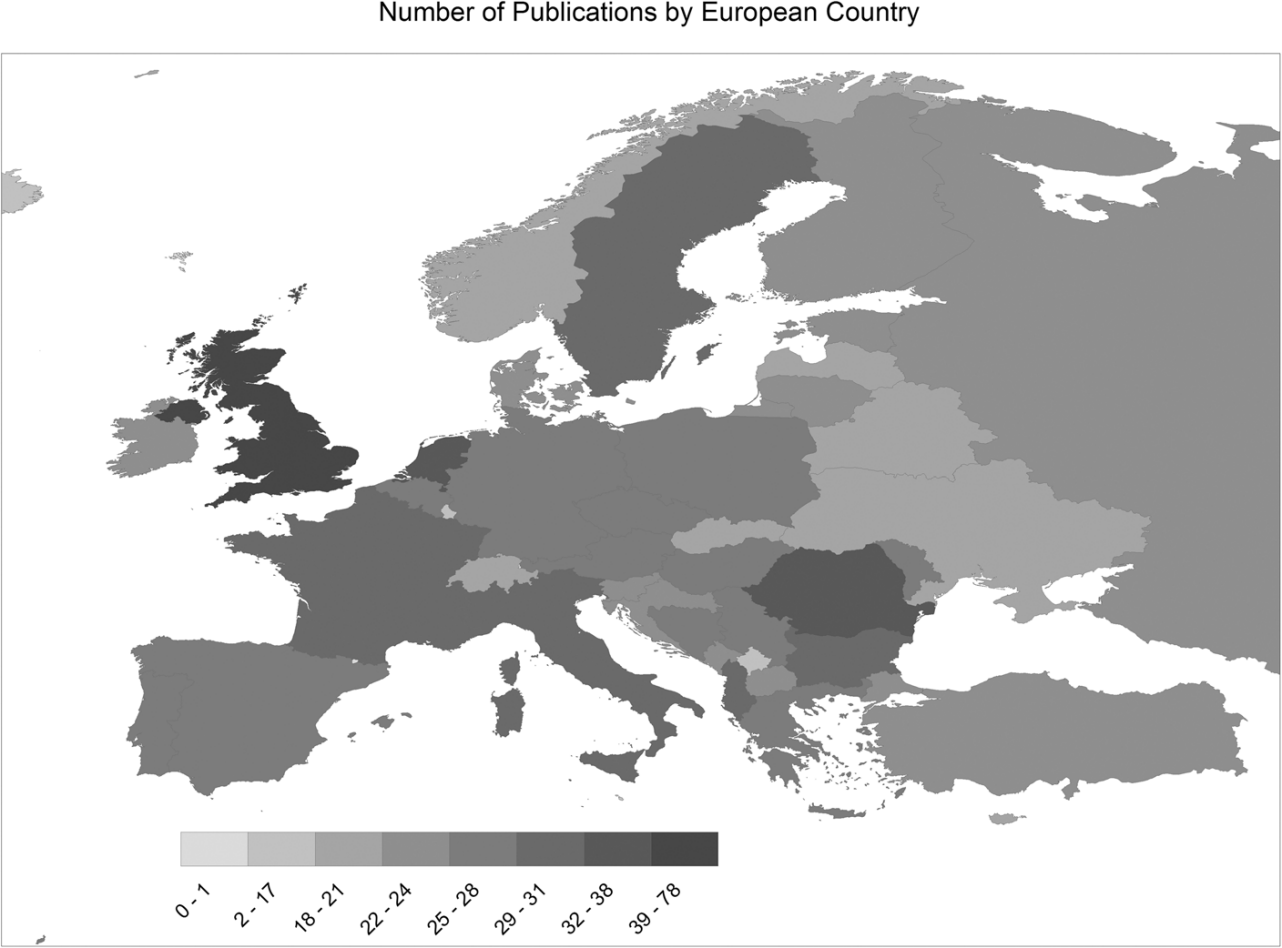
Number of publications by European country
  Publications were split roughly evenly between those dealing with one country (51%, *n* = 78) and multiple countries (49%, *n* = 74). There were striking methodological gaps in 9% of publications (*n* = 13) around precisely which countries were covered in all or part of their enquiries. Finally, multiple-country enquiries generally dealt with each country in isolation and international comparative analyses were rare: notable exceptions include Rijken (2011) and Jokinen and Ollus (2011).
2. Reports dominate the literature and official agencies produce much of the evidence
  There were remarkably few journal articles in the sample: just 12% (*n* = 18). Reports were by far the most common publication format, accounting for 77% (*n* = 117) of the sample. The remainder was comprised of books or book chapters (5%, *n* = 8), theses (1%, *n* = 2) and miscellaneous formats (5%, *n* = 7). We noted that reports were often extremely long: the median was 84 pages (interquartile range of 112) and the longest ran to 644 pages.
  A related issue was publication in non-indexed locations; although the database searches yielded the vast majority of our initial results (87%, *n* = 5340), they contributed just 25% (*n* = 38) of the publications mapped. Most (74%, *n* = 113) came instead from backwards searches, snowballing and requests to stakeholders.
  To understand more about which agencies shape the trafficking knowledge base, we assessed documents’ authorship (Fig. 5). Just 26% of publications came from academia, compared to 54% from governmental and intergovernmental agencies combined.

**Fig. 5:**
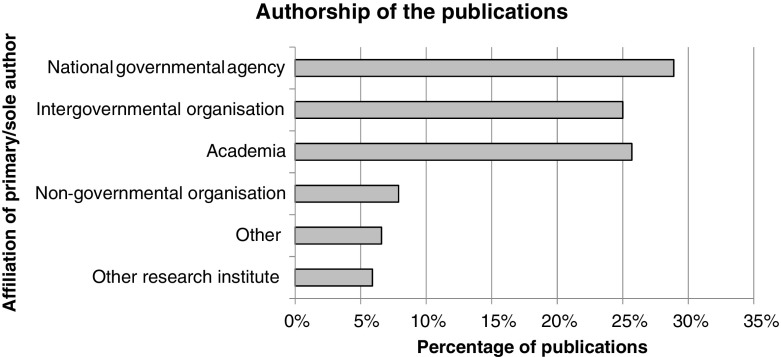
Authorship of the publications
3. There is a striking lack of scientific research
  Figure 6 shows the proportion of publications to contain each of the different types of enquiry. These modes of enquiry were not mutually exclusive; indeed almost a third of publications (31%, *n* = 47) featured the combination of a literature review and other non-scientific enquiry.

**Fig. 6:**
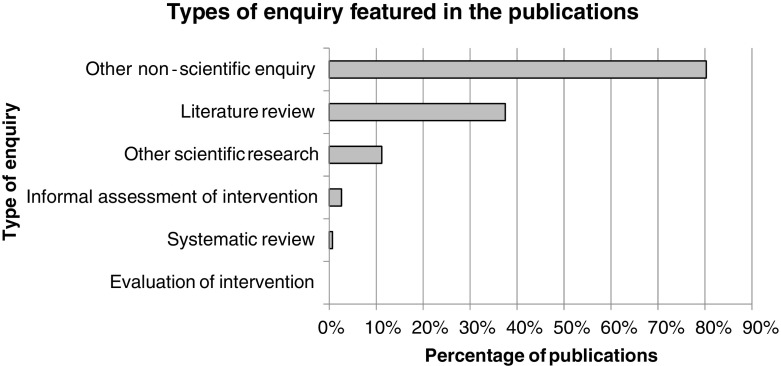
Types of enquiry featured in the publications
  Only 12% (*n* = 18) of publications contained anything that met our definition of scientific research, despite the standards for this being fairly rudimentary (see Fig. 2). Of course, not all publications in the map were presented as containing ‘research’: for example, many summaries of administrative data produced by law enforcement agencies were not (e.g. National Crime Agency 2015; National Criminal Police 2007; National Police Board 2009, 2010, 2011, 2012; Serious Organised Crime Agency 2011). Numerous publications were, however, explicitly framed as research yet lacked the basic characteristics of scientific enquiry; examples with academic authors included Degirmencioglu et al. (2008), Oude Breuil (2008), Pearce et al. (2009) and Ryazantsev et al. (2015). Such issues were also found in reports from official agencies. A key example was the U.S. State Department’s Trafficking in Persons Reports, an influential series of publications. The extremely limited and vague information on methods, data and analysis they contained sat uneasily with their bold claims about ‘a rigorous methodology’ (U.S. Department of State 2001), data from ‘credible reporting’ (U.S. Department of State 2001, 2002, 2003) and being the ‘gold standard’ in assessing anti-trafficking responses (U.S. Department of State 2013).
  Insufficient methodological transparency and a lack of analytical rigour were common shortcomings in the mapped publications. Examples include a methods section that merely listed data sources (Organization for Security and Co-operation in Europe 2014) and an interview-based study that did not report on the number of participants, their characteristics or the questions asked (International Centre for Migration Policy Development 2010b). Another frequent issue was inferences based on weak or inappropriate evidence, for example, generalisations exceeding what could reasonably be concluded from the methods and data (Anti-Slavery International 2014) or unsubstantiated claims of causality (Equality and Human Rights Commission 2011).
4. Qualitative approaches far outweigh quantitative ones
  Far more of the publications were exclusively qualitative (43%, *n* = 65) in design than exclusively quantitative (15%, *n* = 22). Although mixed methods enquiries were common (32%, *n* = 49), within them, qualitative methods dominated and the quantitative contribution rarely exceeded the description of basic numerical data. Again highlighting the methodological weaknesses of the overall evidence base, 11% (*n* = 16) of publications contained insufficient information even to establish whether the design was qualitative, quantitative or both.
5. There are major gaps in coverage around the impacts of labour trafficking itself and counter-measures
  Publications frequently contained a description of the problem profile of labour trafficking (89%, *n* = 135) and/or a discussion of interventions (66%, *n* = 98).^6^ Yet, very few included assessments of the impacts of either labour trafficking (7%, *n* = 10) or of interventions (3%, *n* = 4). Five of those that assessed labour trafficking’s impacts met our criteria for scientific research, including a small but noteworthy set of studies addressing health impacts (Oram et al. 2012a, b; Turner-Moss et al. 2014). Publications involving assessments of interventions, none of which met our criteria for a scientific evaluation, focused either on victims’ perceptions of interventions (International Centre for Migration Policy Development 2007, 2010a) or practitioners’ perceptions of barriers to interventions (The Anti-Trafficking Monitoring Group 2010, 2012).

### Phase 2: Detailed synthesis

We synthesised eight studies identified as key scientific research evidence on European labour trafficking. We report here on the studies’ design (Table 4) and their key findings, conclusions and recommendations (Table 5). Table 5 also includes the studies’ overall quality assessment scores and some general comments on the strength of the evidence they contain. Appendix 7 contains a full breakdown of their scores across the quality assessment criteria.

**Table 4:** Design of the synthesised studies (*n* = 8)

Authors and year	Key foci	Countries covered	Nature of data used	Methods used	Study design	Sampling	Overall sample	Definition of labour trafficking
Antal and Laszlo 2015	Exploration of nature of labour trafficking within and from Romania, in particular overall trends, risk factors, stages in the trafficking process, traffickers’ control strategies and how victims seek help.	Romania	Primary	Qualitative	Interviews	Sampling for areas with high density of victims as sites to interview professionals. No further information about sampling of interviewees who were professionals and none about those who were victims.	Semi-structured interviews with 16 professionals and 7 victims of labour exploitation.	No definition of (labour) trafficking provided.
Gavra and Tudor 2015	Exploration of social and institutional factors behind international trafficking of Roma and of ways of combatting it; analysis covers victims’ characteristics, stages in the trafficking process, offender group structure, authorities’ involvement in the crime and approach to investigation and prosecution.	Romania	Secondary	Qualitative	Documentary analysis of a case study	Case said to be representative of trafficking in Roma (reasons cited include number of people, duration of offending and complexity of investigation), but no substantiating evidence provided.	Analysis of indictments and judgements from the ‘Ţăndărei’ case: a major investigation in which 24 offenders were indicted for international trafficking (to the UK in particular) of Roma children from the Ţăndărei area between 2002 and 2010.	United Nations (2000) for human trafficking.
Jokinen and Ollus 2011	Description of nature and extent of labour trafficking and responses to it, including an exploration of the overlap with labour exploitation more broadly; development of a method for systematising future collection of labour trafficking research data.	Estonia, Finland, Poland	Primary and secondary	Qualitative	Interviews, case records, expert meetings	Sampling of participants for diversity and expertise. Use of administrative data.	Interviews with 40 experts and 13 victims of labour exploitation; records from 32 court cases; pre-trial investigative material from 4 cases; information from 6 expert meetings.	United Nations (2000) for human trafficking. ILO Conventions 29 and 105 for forced labour. Domestic servitude implicitly counted as labour trafficking.
Oram et al. 2012b	Assessment of physical health impacts of trafficking reported by female victims; analysis of factors associated with variations in prevalence of symptoms.	Moldova	Primary	Quantitative	Survey interviews	Consecutive sampling	120 internationally trafficked women (including 23 who were labour-trafficked) supported by the International Organisation for Migration on return to Moldova.	United Nations (2000) for human trafficking. Domestic servitude explicitly counted as labour trafficking.
Rijken 2011	Assessment of current practice, obstacles and good practice in identifying victims of international labour trafficking, investigating and prosecuting cases.	Austria, Netherlands, Romania, Serbia, Spain	Primary and secondary	Qualitative	Interviews, case study analysis	No information provided	Interviews with ‘at least’ 10 experts per country but no information on size of overall sample nor its other characteristics; 10 case studies.	United Nations (2000) for human trafficking. Domestic servitude implicitly counted as labour trafficking.
Tamas et al. 2013	Assessment of trafficking for begging, focusing on victims’ and offenders’ risk factors for victimisation and measures that might help with prevention.	Romania	Primary and secondary	Qualitative and quantitative	Interviews, case records	Use of administrative data. No information provided on sampling of interviewees	Interviews with 20 experts and 8 victims; data on 191 victims from national trafficking database; Information on 123 suspects.	United Nations (2000) for human trafficking. Begging explicitly defined.
Turner-Moss et al. 2014	Description of living and working conditions, prevalence of abuse and physical and mental health symptoms among male and female victims of labour trafficking.	UK	Secondary	Quantitative	Case records	Consecutive sampling	Information from health intake assessment forms for 35 labour trafficking victims supported by Migrant Help (a non-governmental organisation and service provider)	United Nations (2000) for human trafficking. Domestic servitude implicitly counted as labour trafficking.
UNICEF and Save the Children Norway 2012	Assessment of the nature and extent of child begging and street work, the degree to which it involves trafficking and is organised/forced/ coerced by adults; assessment of current service provision, including its strengths, weaknesses and gaps.	Bosnia and Herzegovina	Primary and secondary	Qualitative and quantitative	Surveys, interviews, focus groups observations	Opportunity sampling	Surveys and follow-up interviews with professionals (size of sample and participants’ characteristics unclear); Interviews with 44 street children; focus groups with 95 adults from general population; observations at 8 research sites.	No definitions of trafficking, begging or street work provided.

**Table 5:** Key evidence from the synthesised studies (*n* = 8) and assessment of the strength of that evidence

Authors and year	Key evidence				Quality assessment	
	Key findings	Main conclusions	Recommendations for research	Recommendations for responses	Score	Comments on strength of evidence
Antal and Laszlo 2015	According to interviewees, labour trafficking is typically overlooked and its severity underestimated, leading to under-identification of victims. Victims come from some of the most vulnerable social groups. Recruitment generally happens through acquaintances but also online advertisements and strangers’ approaches. Type of transportation depends on offenders’ modus operandi. Food and accommodation below minimum living standards. Victims work long hours for little or no pay. Traffickers’ initial approach designed to inspire trust and involves various false promises (good pay, good accommodation etc.); subsequent behaviour designed to scare and isolate victims and deter them from seeking help. Control strategies include threats, humiliation, physical violence and denial of access to telephone. Escape thought to be difficult. Police and other authorities criticised for failing to take initiative, overlooking crimes and being influenced by prejudices against Roma.	Labour trafficking is a complex issue, involving multiple factors. International labour trafficking is a form of failed migration. Labour trafficking is fuelled by increases in illegal work, unemployment, poverty and corruption, lack of information, lack of preventative efforts and the negative effects of certain laws designed to protect vulnerable groups. Groups most at risk include people who are: extremely poor; in foster homes; unqualified; disabled, Roma; children. Compared to women, men are more commonly subject to labour exploitation, especially international labour trafficking. For women, labour trafficking often also combined with sexual exploitation.	None made.	Policies and other responses need to be tailored towards the needs of the poorest and most disadvantaged groups (e.g. Roma, disabled).	5/18	No information provided about type of questions asked and very little information about participants. Results largely presented as empirical fact rather than interviewees’ perceptions/opinion. Conclusions not properly grounded in the results and overextend the data.
Gavra and Tudor 2015	Traffickers were generally unemployed and uneducated (20/24 had completed no formal education). Offender group split into those who handled recruitment and those who controlled harbouring and exploitation. Recruitment took place in poor, rural, Roma communities and impoverished and/or disabled children targeted in particular. Victims aged 8 to 16 years. Around 180 children believed to have been trafficked out of Romania by this group alone. Recruitment involved false promises of financial aid to children’s families or the exploitation of existing debts (which had been deliberately engineered for this purpose). Victims depended on traffickers both physically and psychologically. Control techniques included withholding earnings, linguistic isolation and stopping communication with families. Accommodation crowded and inadequate. Victims treated badly: e.g. threatened, poorly clothed, forced to work in any weather conditions, not properly fed. Exploitation included begging and other antisocial/criminal acts (e.g. washing windscreens, pickpocketing). Eighty of the victims were arrested for crimes in the UK (e.g. fraud, begging, theft, aggression). All earnings claimed by offenders and profits substantial. Financial investigations linked traffickers to large sums of money, mansions, land, luxury cars etc. Offenders all Romanian Roma and crime group organised around clan system with a central dominant family and smaller family groupings. Core family had relationships with local authorities (local police, border police and courts).	Minors (especially disabled ones) particularly vulnerable to trafficking due to physical and cognitive immaturity, lack of judgement and inability to assess risks. Victims’ ethnicity made them easy targets because their families did not protect them, were manipulated or were compliant in the trafficking. Victims at risk of re-trafficking. Trafficked children lost contact with education system, lack qualifications and were subjected to behavioural modelling in deviance. The case highlights the ineffectiveness of the Romanian criminal justice system: it was extremely slow, organised criminals were set free and victims were not provided help with social reintegration. Case reflects widespread corruption and dysfunction of Romanian state institutions and lack of respect for laws, regulations and human rights.	None made.	Focus on reducing vulnerability through improved reintegration of victims, improved cooperation with destination countries, greater engagement with Romanian embassies, capacity building among authorities especially around combatting corruption, more effective management of criminal cases and increased penalties for trafficking.	2/18	Unsubstantiated claim that case is representative of human trafficking in Romania. Far too much generalisation from a single case study. Very little information on methods and none on analysis. Results of variable (but generally low) quality in terms of clarity and strength of supporting evidence: those related to ways of combating trafficking particularly weak scientifically. Suggested interventions not properly rooted in results and clearly overextended from the data. Neither ethics nor limitations addressed.
Jokinen and Ollus 2011	Situations vary between Estonia, Finland and Poland in respect to responses (laws, practices, etc) but labour trafficking itself shows similar traits. Victims typically from (poorer) foreign countries and lack language skills. Victims typically the same nationality as offenders. Recruitment involves deception. Victims often indebted prior to arrival. Victims exploited across diverse sectors. Living and working conditions poor and exploitation often sufficiently bad to meet indicators for forced labour. Victims rarely physically confined and subtler forms of coercion/control are more common than direct violence. Barriers to victim identification and support provision include fear of deportation, reluctance to report etc. Involvement of organised crime groups varies between the countries studied. Criminal investigations have proved lengthy and complex. Labour trafficking is a hidden issue and official statistics underrepresent its true scale.	Results highlight an overlap in practice between labour trafficking and other forms of labour exploitation. Caution against attempts to delineate too absolutely between these issues – they occupy a continuum. Findings correspond with research into labour trafficking in other European countries. Current services are focused on sex trafficking and may inadequately meet labour trafficking victims’ needs. Another major conclusion/output was the development of a method for descriptive research into labour trafficking/ exploitation.	Exit surveys among migrant workers leaving Finland and surveys of returned migrant workers in Estonia and Poland, focusing on experience of exploitation.	Improve awareness of labour trafficking among public and professionals. Provide training for professionals on indicators. Improve service provision for labour trafficking victims. Increase resourcing and political prioritisation around tackling labour trafficking. Develop national referral mechanisms. Implement National Rapporteurs in Estonia and Poland. Implement in Finland guidelines on interpreting definition of labour trafficking. Clarify distinction in practice between forced labour, labour exploitation and labour trafficking in law in Poland. Implement specific anti-labour trafficking law in Estonia. Increase efforts to undercover illegal recruitment and facilitation in Estonia.	9/18	Multi-country summary report clear and well-written but based on component country studies of variable quality, reflected in a reduced score. Particular issues with results and conclusions sections of individual country reports, which were very lengthy and of variable quality, lacking in places clarity, coherence and substantiation. These shortcomings limit confidence in validity of overall results/conclusions. Limitations not addressed. Finally, the new method proposed for descriptive research lacked sufficient detail of analytical techniques.
Oram et al. 2012b	Participants ranged from 18 to 44 years (mean 25.4, SD 5.97). 81% (*n* = 97) trafficked for sex, rest for labour. Only limited findings disaggregated by trafficking type. Comparison of reported symptoms for sex versus labour trafficking victims suggests the two may have different health profiles – but bivariate logistic regression gave non-significant results. Study said to be underpowered to detect differences in health impacts between sex and labour victims. High prevalence in the overall sample of diverse symptoms, including neurological ones (headaches: 62%, *n* = 74), gastrointestinal (stomach pain: 61%, *n* = 73) and musculoskeletal (back pain: 44%, *n* = 51; tooth pain: 35%, *n* = 42).	Female trafficking victims returning home may suffer diverse physical and psychological problems. Living and working conditions may contribute to health risks of trafficking. Findings for sample as a whole correspond with previous research into sex trafficking and domestic abuse (although no details were given of prevalence of symptoms in general population).	Further research into health impacts of trafficking, using a larger sample to investigate possible differences associated with sociodemographic factors and characteristics of trafficking experience (including sex vs. labour trafficking).	Provide more comprehensive health services for trafficking victims, including psychological support. Expand focus of policies and programmes beyond sex trafficking alone. Improve recognition of and provision for victims of labour trafficking, whose support needs may differ from sex trafficking victims.	17/18	Excellent exploratory study into under-researched topic. Robust, transparent and appropriate design and clear reporting. Limited amount of labour trafficking-specific findings. Biggest issues are small sample size (especially of labour trafficking victims) and generalisability of findings. Limitations made explicit.
Rijken 2011	Considerable variation between countries studied. Confusion around what constitutes labour trafficking. Difficulties drawing clear distinction between illegal working, labour exploitation and trafficking. Problems with non-recognition and referral of labour trafficking victims (including trafficked persons own perceptions/self-identification as victims). Differences in needs and characteristics of labour and sex trafficking victims. Involvement of more agencies in counter-measures hampered by factors such as limited awareness, investigative powers or familiarity with law. Inadequate information exchange between agencies (including internationally), whereby barriers to improved collaboration include trust, data protection etc. Under-use of pan-European agencies (e.g. Europol).	Trafficking has mainly been associated with sexual exploitation; attention to labour exploitation more recent. Existing provisions geared towards sex trafficking and female victims. Lack of provisions for labour trafficking victims. Insufficient clarity and consensus around what constitutes labour exploitation. Labour trafficking cases hard to identify/misidentified as illegal immigration.	Research into practitioners’ reluctance to make use of Europol and Eurojust as tools for international collaboration. Research into how victim assistance services can complete applications for reflection periods and residence permits.	Develop EU-wide indicators for labour trafficking to help identify cases and understand employment practices to avoid. Improve information sharing nationally and at EU-level. Change migration policies to be more inclusive of less qualified migrants. Give non-law enforcement agencies a more central role in victim identification and support. Enhance existing regulation and monitoring of labour sectors to be more sensitive to trafficking. Awareness raising for diverse groups (potential victims, professionals, employers etc.). Improve support services for victims. Reduce reliance on victim testimony in court.	5/18	Heavily descriptive work. Almost complete reporting void around methods. Methodological opacity a major barrier to assessing the strength of the design, findings and conclusions. Recommendations not always properly grounded in results. Limitations not addressed.
Tamas et al. 2013	Focus on trafficking for begging. Of the 191 identified victims (9% of all identified human trafficking victims in Romania in 2010 and 2011), 79% were adults. Males accounted for 70% of adult victims and 60% of child victims. 81% of victims trafficked internationally. 31% recruited by strangers. Wide range of groups labelled high risk (poor, elderly, children, people with disabilities). Victims’ pre-trafficking lives characterised by extreme poverty. Adult victims typically offered job abroad, children the opportunity to beg. Victims solicited money through diverse forms of begging. Long working hours (up to 18 hours/day in extreme cases). Control mechanisms varied and included threats, violence, supervision, limited freedom of movement and confiscation of identification documents. Limited and substandard food, shelter and clothing provided. Of the 123 suspects investigated, majority (91%) implicated in international trafficking. 48% of international trafficking suspects linked to organised groups.	Victims are a heterogeneous group. Need for improved identification and referral of victims. Diverse initiatives needed, including fundamental measures to reduce vulnerability of at-risk groups.	Research into the characteristics of offenders who traffic victims internally for forced begging. Research into factors making Roma groups vulnerable to trafficking for forced begging.	Improve protection and integration of marginalised groups. Increase public awareness of trafficking for begging. Increase penalties for traffickers. Criminalise begging in all European countries and increase police presence. Improve protections for existing victims and early interventions. Improve training for professionals. Increase interagency and international co-operation.	6/18	Ambitious multi-method exploratory study but needs more rigour and clarity. Gaps and inconsistencies in methods. Results not presented in clear and easily digested fashion and key metrics and/or sources sometimes missing. Findings from interviews heavily overextended from the underlying data. In contrast, results based on analysis of 191 victims much more robust. Overall, results do not comprehensively speak to the original questions posed. Recommendations not well grounded in results. Limitations only partially addressed.
Turner-Moss et al. 2014	Participants typically male (77%, *n* = 27) and aged 21 to 35 years (mean 32.9, SD 10.2, range 19–56). Majority (81%, *n* = 25) reported one or more symptoms of poor physical health. Most common symptoms included headache (43%), back pain (35%), fatigue (30%), vision problems (23%) and dental pain (23%). High proportion (exact % unclear) reported poor working and/or living conditions. 40% (*n* = 12) subject to physical violence; Levels of depressive and anxiety symptoms high. 57% (*n* = 17) reported one or more symptoms of post-traumatic stress.	Men and women who have recently exited labour trafficking situation are likely to have a range of physical and mental health problems needing attention. Findings on physical health impacts correspond with previous research among female victims of trafficking for sexual and labour exploitation. Industries in which labour trafficking victims were exploited are associated with health problems/risks: trafficking may heighten general occupational hazards.	Further research into health impacts of labour trafficking, using larger sample and exploring association with sociodemographic and other factors and addressing responses to intervention. Research into health risks/problems experienced by trafficked versus non-trafficked workers in specific labour sectors.	Improve recognition of health impacts of labour trafficking. Extend provision of health assessments and forensic medical examinations for victims. Provide training for healthcare professionals to help recognise signs of labour trafficking and to respond to complex physical and psychological needs of victims in post-trafficking situations.	16/18	Strong exploratory study into under-researched topic. Transparent and appropriate design and clear reporting. Small sample size and lack of comparison groups pose barriers to generalisability. Limitations made explicit.
UNICEF and Save the Children Norway 2002	Substantial number of children living or working on the streets, including a disproportionately high number of Roma. Numerous concerns for street children’s safety and well-being, but proportion of cases involving trafficking unclear. Limited awareness and understanding of trafficking. Numerous barriers to intervention, including limited co-operation between agencies and insufficient resources.	Distinction between labour trafficking and exploitation more broadly is not clear-cut - and notably less so than for sex trafficking. Extent of trafficking unclear. Street children face extensive problems in general. Current responses are inadequate.	Research into child labour more broadly in Bosnia and Herzegovina. Action oriented research with Roma community’s participation to explore ways of protecting Roma children from exploitation, neglect and abuse.	Develop coherent response to child labour, ensuring access to support services not contingent on meeting trafficking definition. Focus on different aspects to response (law enforcement, social work etc.) depending on level of criminal organisation/family involvement etc. Improve recognition of victims’ vulnerability and ensure they are not treated as offenders. Improve awareness and tackle current stereotypes and prejudices. Improve general protective systems and service provision for children in Bosnia and Herzegovina.	3/18	Study deals primarily with child begging/street work, with a limited amount of labour trafficking-specific findings. Ambitious multi-method exploratory study that lacks scientific rigour and methodological transparency. Incomplete and conflicting methodological information. Major issues with coherence and clarity in reporting in general and substantiation of results in particular. Validity of results and conclusions hard to assess. Limitations only partially addressed. Very little on ethical considerations of research regardless of focus on children.

For multi-country studies, we detail the overall samples from European countries and, unless explicitly stated otherwise, findings and conclusions relate to all European countries involved. Some of the synthesised studies also included data, methods and/or topics outside our review’s stated remit: for example, Rijken (2011) contained an analysis of policy and law around labour trafficking and UNICEF and Save the Children Norway (2002) had a distinct component addressing child sex trafficking. In such cases, we report only on those elements of the studies concerned with empirical evidence on European labour trafficking.

To complement the results at individual study level, we now consider some overarching findings about the synthesised studies’ design, quality and contents.

1. There were some commonalities in design
  First, all eight synthesised studies were exploratory in design, reflected in broad and inclusive foci and a predominance of descriptive research. The two studies into trafficking’s health impacts (Oram et al. 2012b; Turner-Moss et al. 2014) were, however, notably more focused than the others in their questions, methods and analysis.
  Second, all studies used non-random sampling. This is particularly noteworthy for those studies involving quantitative enquiries (*n* = 4) since it limits their external validity.
  Third, certain methods were especially common: semi-structured interviews and analyses of case records were used in five studies apiece.
  Fourth, there were some commonalities in definitional approaches. Six of the eight studies defined human trafficking with explicit reference to the ‘Palermo Protocol’ (United Nations 2000); the two exceptions (Antal and Laszlo 2015; UNICEF and Save the Children Norway 2002) gave no definition. Three studies focused on the same specific sub-set of labour trafficking: begging/street work (Gavra and Tudor 2015; Tamas et al. 2013; UNICEF and Save the Children Norway 2002). The other five dealt with labour trafficking as a broad category (Antal and Laszlo 2015; Jokinen and Ollus 2011; Oram et al. 2012b; Rijken 2011; Turner-Moss et al. 2014). Of the latter, none provided an explicit definition of labour trafficking but all bar one (Antal and Laszlo 2015) implicitly counted domestic servitude as a form thereof.
2. The quality of evidence varied but was often low
  All eight studies met our rudimentary criteria for scientific research applied at the mapping stage. The detailed quality assessment exercise highlighted enormous variation between the studies in terms of their overall calibre, which has obvious implications for the strength of the evidence they contain. Scores out of 18 ranged from a low of 2 (Gavra and Tudor 2015) to a high of 17 (Oram et al. 2012b). The overall quality was low: just three studies scored 50% or higher on the quality assessment. The two top-scoring publications stood out for their methodological transparency and rigour, frank discussion of limitations and clear and concise reporting (Oram et al. 2012b; Turner-Moss et al. 2014).
  At the other end of the spectrum, low scores were often driven by methods reporting that was disjointed, inadequate and opaque, or, in some cases (Gavra and Tudor 2015; Rijken 2011), virtually non-existent. Methodological weaknesses included contradictory information (Tamas et al. 2013); missing information on fundamentals like sample size (Rijken 2011; UNICEF and Save the Children Norway 2002); and incoherence, with key methods information scattered across sections including results (UNICEF and Save the Children Norway 2002). Other drivers of low scores were unclear or imprecise reporting of results and the presentation of conclusions that had little to no grounding in the study data. Illustrative examples include not specifying the empirical basis for various findings despite using multiple methods and datasets (Tamas et al. 2013; UNICEF and Save the Children Norway 2002), and overextending results through presentation that exaggerated their generalisability (Gavra and Tudor 2015; Jokinen and Ollus 2011; Tamas et al. 2013; UNICEF and Save the Children Norway 2002). Taken to the extreme, in one publication, a single case study was framed as representative of an entire country’s trafficking problem (Gavra and Tudor 2015).
3. There were some common themes around labour trafficking and responses to it
  Despite differences in the studies’ foci and design, we identified common themes in their results and conclusions. In reading these findings, readers should be mindful of the variable quality of the original studies.
  - Responses to labour trafficking vary between countries but are generally seen as inadequate
    There was considerable variation between specific European countries in terms of how they conceptualise and respond to labour trafficking and levels of awareness of the problem (Jokinen and Ollus 2011; Rijken 2011). Existing counter-trafficking measures were seen to be too heavily focused on sex trafficking at the expense of labour trafficking (Antal and Laszlo 2015; Jokinen and Ollus 2011; Oram et al. 2012b; Rijken 2011). Current responses to labour trafficking (either in general or for begging/street work in particular) were perceived to be inadequate, including in respect to victim identification, service provision, law enforcement and preventative activity (Antal and Laszlo 2015; Gavra and Tudor 2015; Jokinen and Ollus 2011; Oram et al. 2012b; Rijken 2011; Tamas et al. 2013; UNICEF and Save the Children Norway 2002);
  - Labour trafficking affects diverse sectors and there may be spatial, temporal and sociodemographic trends to consider
    As well as begging and street work (Gavra and Tudor 2015; Tamas et al. 2013; UNICEF and Save the Children Norway 2002), other sectors identified as affected by labour trafficking include food-processing, agriculture, restaurants, nail bars and car washes (Antal and Laszlo 2015; Jokinen and Ollus 2011; Oram et al. 2012b; Rijken 2011; Turner-Moss et al. 2014).
    In terms of spatial, temporal and socio-demographic trends in labour trafficking, there were limits to what could be concluded – especially beyond the level of individual studies. There was evidence to suggest the industries in which labour trafficking is most commonly encountered vary by country (Jokinen and Ollus 2011; Rijken 2011), likely reflecting differences in local labour markets. Temporal patterns were hinted at where labour trafficking affected seasonal industries (e.g. fruit picking) (Jokinen and Ollus 2011). There were also indications that different types of victims may be preferred in different contexts. For example, trafficking of children and/or adults for begging was *not* associated with a pronounced gender imbalance (perceived or actual) (Gavra and Tudor 2015; Tamas et al. 2013; UNICEF and Save the Children Norway 2002). In contrast, men were often reported to be at greater risk of labour trafficking in general than women (the reverse is true of sex trafficking) (Antal and Laszlo 2015; Jokinen and Ollus 2011; Rijken 2011) and study samples of adult labour trafficking victims were indeed predominantly male (Tamas et al. 2013; Turner-Moss et al. 2014).
    Apart from gender, many individual-level characteristics reported to be linked with vulnerability to labour trafficking were related to marginalisation, for example, by merit of being Roma, very young or very old, impoverished, unemployed or working on the street, homeless, in or just out of the care system, an illegal immigrant, and/or disabled (unlike in most other work contexts, disabilities may be advantageous for begging) (Antal and Laszlo 2015; Gavra and Tudor 2015; Jokinen and Ollus 2011; Rijken 2011; Tamas et al. 2013; UNICEF and Save the Children Norway 2002).
  - Labour trafficking victims are poorly treated and commonly display signs of ill health
    Labour trafficking victims were or were perceived to be subject to very poor living and working conditions and to be manipulated through diverse control mechanisms, including threats, violence, withholding pay, confiscation of documents and physical, social and linguistic isolation (Antal and Laszlo 2015; Gavra and Tudor 2015; Jokinen and Ollus 2011; Tamas et al. 2013; Turner-Moss et al. 2014). Symptoms of poor health were high among victims of labour trafficking who accessed support services (Oram et al. 2012b; Turner-Moss et al. 2014). Turner-Moss et al. (2014) found the most commonly reported symptoms of poor physical health were headaches, back pain, fatigue, loss of appetite, toothache or mouth/gum problems, and eye pain, injury or difficulty seeing. They also found high rates of reporting of symptoms of post-traumatic stress, depression and anxiety.
  - Numerous barriers are seen to impede attempts to tackle labour trafficking
    Numerous and diverse barriers to tackling labour trafficking were reported, including inadequate resourcing, limited awareness, unmet training needs, insufficient information sharing, ineffective collaboration, corruption and confusion around what constitutes labour trafficking in the first place (Antal and Laszlo 2015; Gavra and Tudor 2015; Jokinen and Ollus 2011; Oram et al. 2012b; Rijken 2011; Tamas et al. 2013; Turner-Moss et al. 2014; UNICEF and Save the Children Norway 2002).
  - Labour trafficking is seen as a complex, multi-faceted issue requiring holistic responses
    Labour trafficking was characterised as a complex problem that overlaps with numerous other important labour market issues like child labour, forced labour, illegal working, economic migration and occupational health (Antal and Laszlo 2015; Jokinen and Ollus 2011; Rijken 2011; Turner-Moss et al. 2014; UNICEF and Save the Children Norway 2002). It was suggested interventions would benefit from being more systematic and coordinated and better integrating related phenomena (Jokinen and Ollus 2011; UNICEF and Save the Children Norway 2002). In particular, it was argued that protective measures to support vulnerable and exploited individuals should not be contingent on the ‘trafficking’ label being applied: in practice it can be difficult to distinguish between ‘labour trafficking’ and other forms of forced and exploitative labour (Jokinen and Ollus 2011; UNICEF and Save the Children Norway 2002).

## Discussion

We start by setting out the limitations of the study. We then discuss the results and implications for each of the mapping and synthesis stages in turn. Finally, we consider the review’s overarching implications for future research into and responses to labour trafficking.

### Study limitations

There were several potential sources of bias at the review level. First, restricting our review to publications in English may introduce language bias. English is said to be the lingua franca for trafficking research (Laczko and Thompson 2000). It was striking that we excluded just 26 publications for not being in English, accounting for less than 1% of overall exclusions.

Second, there may be publication bias or bias in the identification of studies. Systematic reviews are arguably more challenging in the social than medical sciences since there are more databases with lower indexing standards and publications in non-indexed locations play a greater role (qualitative research is generally less likely to be published in indexed locations) (Brunton et al. 2012; Thomas and Harden 2008). Research from developing countries may be less likely to be published in indexed journals (Zielinski 1995), possibly affecting in particular the coverage of some Eastern European countries in our review. As safeguards against such biases, we searched a broad range of databases (academic and grey literature) and also used diverse complementary search strategies (e.g. asking for recommendations from stakeholders from across Europe).

Third, there may be some coding bias. For clarity and transparency’s sake, we include all coding frameworks in our appendices. Where we single-coded (screening and mapping stages), we made sure to pilot our codebooks, assess for inter-rater reliability (results showed good consistency) and encourage the coders to seek a second opinion where unsure. We double-coded the quality assessment of the studies in the synthesis. Some of the coding categories in the rest of the synthesis were open-ended and narrative in nature. Although unavoidable, as they are appropriate to the review of qualitative literature, such categories introduce greater subjectivity and with it a risk of bias. For validation and quality control, a second coder reviewed and commented on the main coder’s outputs here.

### The systematic map

Despite increased prioritisation of labour trafficking in European counter-trafficking policy and practice (de Jonge 2005; Eurostat 2013; Jokinen and Ollus 2013; National Police Board 2011; Organization for Security and Co-operation in Europe 2008; Rijken 2011), our results indicate that the evidence-base is underdeveloped: across a 15.5-year review period we found just 152 publications containing empirical data on European labour trafficking. Enquiries used primary and secondary data from a wide range of European countries. Although multi-country studies were common, robust comparative analyses were not, which may be inhibiting the development of a coherent European evidence base and contributing to duplication of research efforts. Comparability could be a contributory factor here due to variations in laws, definitions and available data even within European Union member states (de Jonge 2005; International Labour Office 2009; Organization for Security and Co-operation in Europe 2008; Rijken 2011).

While publication in a journal does not guarantee quality, the peer-review process is widely seen as a quality-control measure that promotes rigour and transparency. Yet, the majority of mapped publications were reports: a format that is generally neither well-indexed nor subject to rigorous quality control. The preponderance of grey literature raises concerns about the visibility, accessibility and quality of the evidence on labour trafficking. Digestibility is another consideration: the tendency towards very lengthy publications may limit their dissemination, consumption and, ultimately, impact.

The knowledge-production process was dominated by official agencies, with comparatively little input from academics and other independent parties. Of course, different authorships have different strengths. Robust outputs from practitioners, for example, can capitalise on valuable experiential knowledge and hard-to-access data and sources. In contrast, obvious advantages to academic outputs include the expectation of impartiality, rigorous methods training, strong analytical skills and research experience. The scarcity of academic research may reflect difficulties in securing data access, an oft-cited barrier to trafficking research (Feingold 2010; Goodey 2008; Goździak 2008; Tyldum 2010).

The most disparaging feature of the evidence base was the overall lack of quality research on European labour trafficking. Just 12% (*n* = 18) of mapped publications met our basic criteria for scientific research. It seems that the problem is less that there is too little information, although there are certainly major gaps, and more that so much of it risks being lost because it exists in states not conducive to meaningful appraisal and synthesis. Many publications that fell short of the inclusion criteria for our synthesis contained material that was interesting, empirically-rich and informative (e.g., Andrees 2008; Eurostat 2013, 2015; Migrant Rights Centre Ireland 2007; Surtees 2007, 2008, 2014). Yet, it is difficult to make good use of such information in a review when the methods by which it was originally obtained are weak or unclear.

The predominance of qualitative techniques may well be related to the evidence base’s underdevelopment: less mature fields may gravitate towards exploratory enquiries for which qualitative methods may be optimum. It has also been argued that the quality and quantity of official datasets on labour trafficking are far lower than those on sex trafficking (the longstanding focus of counter-trafficking efforts) (International Labour Office 2009; International Organization for Migration 2010; Organization for Security and Co-operation in Europe 2008). There may also be a reluctance to use official data because of their biases (such as systematic under-reporting). Yet, if the evidence base on European labour trafficking is to progress, there is a also need for robust quantitative enquiries. The current scarcity of quality quantitative research leaves a void that risks being filled by dubious and unreliable statistics.

Numerous publications described the problem of labour trafficking and somewhat fewer the responses to it. Yet, few addressed the impacts of labour trafficking and fewer still the impacts of responses. Understanding the nature of the problem is an important first step, akin to the scanning stage in the SARA (scanning, analysis, response, assessment) model of problem-oriented policing (Eck and Spelman 1987). If the counter-trafficking field is to evolve, it is vital to evaluate the impacts of trafficking and of interventions. The fact that we found no scientific evaluations precludes any conclusions about ‘what works’ or ‘best practice’ in tackling European labour trafficking.

### The synthesis

Traditionally, trafficking research and responses have focused heavily on sex trafficking and concerted interest in labour trafficking is fairly recent (Andrees and Linden 2005; Home Office 2007; Kelly 2005; Laczko and Gozdziak 2005; Surtees 2008). Indicative of the immaturity of the scientific research base on European labour trafficking, just eight studies qualified for inclusion in our synthesis and all bar one of them (UNICEF and Save the Children Norway 2002) were published after 2010.

In light of the literature’s underdevelopment, it was unsurprising to find all the synthesised studies were exploratory and descriptive. All involved non-random sampling, too. Sampling for expertise and/or diversity is fairly standard practice when conducting expert interviews, which was a key method in five of the eight studies (Antal and Laszlo 2015; Jokinen and Ollus 2011; Rijken 2011; Tamas et al. 2013; UNICEF and Save the Children Norway 2002). Four of the eight studies were partially or fully quantitative in design, however (Oram et al. 2012b; Tamas et al. 2013; Turner-Moss et al. 2014; UNICEF and Save the Children Norway 2002) and in this context opportunity sampling clearly limits external validity. It should be emphasised though that traffickers and their victims are hidden populations; insufficient knowledge of their characteristics impedes the creation of reliable sampling frames (Tyldum and Brunovskis 2005). Additionally, trafficking is a high-severity, low-frequency crime (in contrast to ‘volume crimes’ like burglary) and to detect enough cases research samples of the general population might have to be prohibitively large.

Unlike in medicine (Shamseer et al. 2015), systematic reviews in criminology do not routinely include a quality assessment (Johnson et al. 2015). Our quality assessment exercise proved valuable in highlighting the variable strength of evidence (studies’ scores ranged from 2 to 17 out of 18) and weak overall quality (5 out of 8 scored below 50%). These results caution against reporting evidence in a review without also considering its strength. The two highest-scoring publications by far were also the only journal articles (Oram et al. 2012b; Turner-Moss et al. 2014), possibly reflecting the additional quality assurance the peer-review process can bring. 

We found numerous weaknesses in the design, conduct and reporting of other studies in the synthesis: in particular, insufficient clarity and transparency around methods, overextending the data and not paying due consideration to limitations, and conclusions that were not well-grounded in the results. These thematic findings should be treated as preliminary rather than conclusive or exhaustive. Taken as a whole, they suggest that there is a clear and unmet need for improved responses to European labour trafficking, including around prevention, intervention, monitoring and victim support. The possibility of gendered differences in trafficking types (labour trafficking disproportionately affecting men) merits further attention. The severity of labour trafficking was reflected both in its association with symptoms of ill health and more general accounts of extremely poor living and working conditions. While it remains to be seen whether and how the experiences and impacts of labour trafficking diverge from, say, sex trafficking, these results speak to a clear need for investment in both support services for victims of labour trafficking and targeted preventative activity. Numerous perceived barriers to intervention were highlighted, ranging from limited awareness to unmet training needs and widespread corruption. While their actual impact was not assessed, it would be useful to consider these factors when designing and evaluating interventions. The results suggest labour trafficking can affect a diverse range of legal and illegal industries. A more differentiated approach to researching and responding to labour trafficking that takes into account similarities and differences between sectors could prove useful in future. Finally, the overlaps between labour trafficking and other labour market issues (child labour, economic migration, etc.) highlight the importance of situating research on and responses to labour trafficking within a broader spectrum of interrelated matters: labour trafficking should not be reduced to a criminal justice issue alone (see also Dutch National Rapporteur 2009; Esson 2015; Jokinen and Ollus 2011, 2013; Lewis et al. 2014; Skrivankova 2006; UNICEF and Save the Children Norway 2002).

### Overall implications for research and responses

Our criticisms of the literature, in particular the low reporting standards, are not mere ‘academic pedantry’. These shortcomings represent genuine barriers to building and advancing a strong evidence base. Many of our criticisms echo those made in previous (non-systematic) reviews of and commentaries on other aspects to the human trafficking literature. Similar complaints about methodological opacity, lack of rigour, failure to acknowledge and account for limitations and skews in focus and methods have been heard repeatedly for over a decade (Andrees and Linden 2005; Aronowitz 2009; Di Nicola 2007; Feingold 2010; Goodey 2008; Kelly 2005; Laczko 2005; Laczko and Gozdziak 2005; Tyldum and Brunovskis 2005). As Kelly (2005, p. 237) argued, the ‘lack of methodological transparency provides little foundation for assessing the depth and quality of research and denies the entire field opportunities for learning and knowledge transfer.’

As labour trafficking continues to ascend the policy and practice agenda, greater investment in research and interventions will likely follow. It is imperative to avoid repeating previous mistakes, as despite considerable spending on measures to counter human trafficking (especially sex trafficking), responses have rarely been evidence-based and the literature remains notoriously weak (see, e.g., Gozdziak and Bump 2008). Based on our findings, we would particularly recommend the following measures. First, the commissioning of research to address particularly neglected areas, including the impacts of labour trafficking, evaluations of interventions and comparative analyses. Second, increased investment in research that is explanatory rather than purely descriptive; there is a particular need for strong quantitative research that exploits existing datasets or makes use of innovative new datasets. Third, increased academic involvement in empirical research, especially through collaborations between independent researchers and those who act as gatekeepers to data and participants. Finally, but most importantly, overall improvements in research design and reporting standards. This last recommendation is particularly critical as it applies to virtually any study. Although making such improvements is primarily the responsibility of individual researchers, funders and end-users can contribute by holding them to account. What constitutes ‘good’ research and reporting is to a certain extent dependent on specific disciplines and methods, but there are certainly common factors. Rather than reinvent the wheel, we refer readers to the quality assessment tool (Appendix 5): its nine questions provide a useful checklist for designing, reporting and critically consuming research. We would add one further point to the list: the provision of an explicit research definition of labour trafficking, including any inclusion/exclusion parameters and how it was operationalised. Otherwise, the lack of definitional clarity and consistency will remain a barrier to comparing studies.

## Conclusions

Despite a marked increase in attention around labour trafficking in Europe, there is a scarcity of high-quality empirical evidence on the problem, its impacts and responses to it. Our review showed the evidence base to be limited, fragmented and subject to skews in thematic focus and methodological design. Few publications met even basic scientific standards and even those that did were of variable and often low quality. Particularly pronounced problems included a lack of methodological transparency and rigour, shortcomings that restrict what conclusions can be drawn from the literature.

Overall, our synthesis indicated that European labour trafficking is a complex and challenging problem to which current responses may be inadequate. The lack of a coherent and robust research base limits the feasibility of evidence-based policy and practice, which is a concern given the threats labour trafficking poses and the resources devoted to tackling it. Amid numerous knowledge gaps around European labour trafficking, it is vital to pay attention to increasing the quality of the evidence, not just the quantity.

## Electronic supplementary material

ESM 1 (DOCX 71 kb)

## Footnotes

1 Although these terms are often used interchangeably, we deliberately stick to ‘human trafficking’ throughout this article. There is an internationally agreed and widely used definition of human trafficking but not for modern slavery.

2 Based on all referrals to the UK’s ‘National Referral Mechanism’ from 1 April 2009 to 30 September 2014. Victims are defined as individuals granted positive ‘conclusive grounds’ decisions.

3 According to the International Labour Organisation Convention on Forced Labour, ‘*forced or compulsory labour* shall mean all work or service which is exacted from any person under the menace of any penalty and for which the said person has not offered himself voluntarily’ (International Labour Organization 1930). Specific exceptions apply, covering military service for example.

4 A screening codebook is a reference tool that provides information to reviewers about how to apply the screening criteria in practice. Our final codebook contained the information in Table 2 plus relevant supporting documentation (e.g. the list of European countries).

5 Eight publications do not feature in this figure: three covered no longer existing countries (Federal Republic of Yugoslavia and Former Federal Republic of Yugoslavia) and five did not specifically cover any European countries. Those not covering any European countries as an explicit source of primary or secondary data nonetheless met the inclusion parameters as they featured empirical data on victims who were themselves from Europe (e.g. Eastern Europeans trafficked to the United States for labour exploitation).

6 These categories were not mutually exclusive so percentages do not sum to 100%.
