# Supplementary material for: Human trafficking for labour exploitation: the results of a two-phase systematic review mapping the European evidence base and synthesising key scientific research evidence
Source: J Exp Criminol. 2018 Apr 6;14(3):319–60. doi: 10.1007/s11292-017-9321-3 (PMC6417370; doi:10.1007/s11292-017-9321-3)
Supplement: Supplementary file 1 — (DOCX 71 kb) [file 11292_2017_9321_MOESM1_ESM.docx]

**Appendices**

**Appendix 1: PRISMA-P checklist**

**PRISMA-P (Preferred Reporting Items for Systematic review and Meta-Analysis Protocols) 2015 checklist: recommended items to address in a systematic review protocol***

| **Section and topic** | **Item No** |  | **Checklist item** | |
| --- | --- | --- | --- | --- |
|  | | | | **ADMINISTRATIVE INFORMATION** |
| Title: |  |  |  | |
| Identification | 1a | ✓ | Identify the report as a protocol of a systematic review | |
| Update | 1b | n/a | If the protocol is for an update of a previous systematic review, identify as such | |
| Registration | 2 | ✓ | If registered, provide the name of the registry (such as PROSPERO) and registration number | |
| Authors: |  |  |  | |
| Contact | 3a | ✓ | Provide name, institutional affiliation, e-mail address of all protocol authors; provide physical mailing address of corresponding author | |
| Contributions | 3b | ✓ | Describe contributions of protocol authors and identify the guarantor of the review | |
| Amendments | 4 | n/a | If the protocol represents an amendment of a previously completed or published protocol, identify as such and list changes; otherwise, state plan for documenting important protocol amendments | |
| Support: |  |  |  | |
| Sources | 5a | ✓ | Indicate sources of financial or other support for the review | |
| Sponsor | 5b | ✓ | Provide name for the review funder and/or sponsor | |
| Role of sponsor or funder | 5c | ✓ | Describe roles of funder(s), sponsor(s), and/or institution(s), if any, in developing the protocol | |
|  | | | | **INTRODUCTION** |
| Rationale | 6 | ✓ | Describe the rationale for the review in the context of what is already known | |
| Objectives | 7 | ✓ | Provide an explicit statement of the question(s) the review will address with reference to participants, interventions, comparators, and outcomes (PICO) | |
|  | | | | **METHODS** |
| Eligibility criteria | 8 | ✓ | Specify the study characteristics (such as PICO, study design, setting, time frame) and report characteristics (such as years considered, language, publication status) to be used as criteria for eligibility for the review | |
| Information sources | 9 | ✓ | Describe all intended information sources (such as electronic databases, contact with study authors, trial registers or other grey literature sources) with planned dates of coverage | |
| Search strategy | 10 | ✓ | Present draft of search strategy to be used for at least one electronic database, including planned limits, such that it could be repeated | |
| Study records: |  |  |  | |
| Data management | 11a | ✓ | Describe the mechanism(s) that will be used to manage records and data throughout the review | |
| Selection process | 11b | ✓ | State the process that will be used for selecting studies (such as two independent reviewers) through each phase of the review (that is, screening, eligibility and inclusion in meta-analysis) | |
| Data collection process | 11c | ✓ | Describe planned method of extracting data from reports (such as piloting forms, done independently, in duplicate), any processes for obtaining and confirming data from investigators | |
| Data items | 12 | ✓ | List and define all variables for which data will be sought (such as PICO items, funding sources), any pre-planned data assumptions and simplifications | |
| Outcomes and prioritization | 13 | ✓ | List and define all outcomes for which data will be sought, including prioritization of main and additional outcomes, with rationale | |
| Risk of bias in individual studies | 14 | ✓ | Describe anticipated methods for assessing risk of bias of individual studies, including whether this will be done at the outcome or study level, or both; state how this information will be used in data synthesis | |
| Data synthesis | 15a | ✓ | Describe criteria under which study data will be quantitatively synthesised | |
|  | 15b | n/a | If data are appropriate for quantitative synthesis, describe planned summary measures, methods of handling data and methods of combining data from studies, including any planned exploration of consistency (such as I^2^, Kendall’s τ) | |
|  | 15c | n/a | Describe any proposed additional analyses (such as sensitivity or subgroup analyses, meta-regression) | |
|  | 15d | ✓ | If quantitative synthesis is not appropriate, describe the type of summary planned | |
| Meta-bias(es) | 16 | ✓ | Specify any planned assessment of meta-bias(es) (such as publication bias across studies, selective reporting within studies) | |
| Confidence in cumulative evidence | 17 | ✓ | Describe how the strength of the body of evidence will be assessed (such as GRADE) | |

***** **It is strongly recommended that this checklist be read in conjunction with the PRISMA-P Explanation and Elaboration (cite when available) for important clarification on the items. Amendments to a review protocol should be tracked and dated. The copyright for PRISMA-P (including checklist) is held by the PRISMA-P Group and is distributed under a Creative Commons Attribution Licence 4.0.**

*From: Shamseer L, Moher D, Clarke M, Ghersi D, Liberati A, Petticrew M, Shekelle P, Stewart L, PRISMA-P Group. Preferred reporting items for systematic review and meta-analysis protocols (PRISMA-P) 2015: elaboration and explanation. BMJ. 2015 Jan 2;349(jan02 1):g7647.*

**Appendix 2: Search terms**

Due to differences in search functionality, different search strings were used for the database searches and the electronic searches of key journals.

The search structure and keywords used for the database searches (title, abstract, keyword basis) was as follows:

(Publication year > 1999)

AND

(“Human trafficking” OR “trafficking in human beings” OR “human traffic” OR “people trafficking” OR “child trafficking” OR “forced labo?r” OR “debt bondage” OR “slavery” OR “trafficker” OR “trafficked”)

AND

((Labo?r)

OR

(Cannabis OR marijuana AND factory OR farm OR cultivat* OR grow*)

OR

(Marriage OR pickpocket* OR theft OR thief OR thiev* OR begging OR beggar OR fraud* OR agricultur* OR fish* OR construction OR paving OR nail OR salon OR farm OR package* OR industry OR factory OR restaurant OR catering OR maritime)).

For the journal searches, we selected search terms that were broad in scope but relatively few in number. This was due to the additional demands of manual searching and limited search functionality of the sources. The following terms were used to search within each journal for publications from 2000 onwards:

- “Human trafficking”
- “Trafficking in human beings”
- “Human traffic”
- “People trafficking”
- “Forced labour”/”forced labor”
- “Labour exploitation”/”labor exploitation”
- “Modern slavery”
- “Debt bondage”

**Appendix 3: Coding framework for the systematic map**

| Category of interest | Variable(s) coded |
| --- | --- |
| Identifying data | - Unique identifying code - Title of publication - Name of author(s) |
| Date of publication | - Year of publication |
| Authorship | - Affiliation of first or only author (*e.g.* academia, non-governmental organisation) |
| Format of publication | - Mode of publication (e.g. journal article, report) |
| Source of publication | - Through which search strategy was this publication located (e.g. SCOPUS, journal searches) |
| Type of enquiry | - Research design (qualitative, quantitative, mixed methods) - Type(s) of enquiry involved (e.g. systematic review, evaluation). See Figure 2 for a breakdown of how these were categorised. |
| Geographical coverage | - Single or multiple country enquiry - If made explicit, the total number and list of European countries included in the research (as sources of primary or secondary data) |
| Focus of publication | - On which aspects to labour trafficking empirical information was provided (categorised as description of problem profile, assessment of impacts of problem, discussion of interventions and assessment of impacts of interventions). |

**Appendix 4: Coding framework for the synthesis**

| Category of interest | Variable(s) coded |
| --- | --- |
| Focus | - What was the hypothesis, research question(s) or statement of intent? |
| Methods | - Study design (e.g. interviews, surveys, experiments) - Primary and/or secondary data - Unit(s) of analysis - Sampling method - Sample size - Characteristics of participants - The specific sub-sectors of labour trafficking, if any, that were covered in the study |
| Definition of labour trafficking | - Study definition of labour trafficking - Whether or not domestic servitude cases were included as labour trafficking |
| Key findings, conclusions and recommendations | - Key findings - Main conclusions - Any recommendations made for a) researching and b) tackling labour trafficking |

**Appendix 5: Quality assessment tool (loosely adapted from Oram *et al.*, 2012)**

| Question | Score |
| --- | --- |
| Does the study have a clear, explicit and feasible research question(s) or statement of intent(s) | Each question scored as follows:  0= Publication does not meet the criterion and/or does not contain sufficient information to assess whether it meets the criterion at all  1= Publication partially meets the criterion and/or only contains information permitting a partial assessment of whether it meets the criteria  2= Publication fully meets the criterion |
| Is the overall study design clear and appropriate? |  |
| Have ethical considerations been explicitly discussed and properly managed? |  |
| Is the sampling method explicit and appropriate? |  |
| Is the sample itself adequate? |  |
| Is the analytical procedure transparent and appropriate? |  |
| Are the results clear and precise? |  |
| Are limitations identified and accounted for? |  |
| Are the conclusions properly grounded in the results? |  |

**Appendix 6: List of publications included in the systematic map**

1. Alexandru, M., and Ene T. (2014). Child Trafficking in Romania, a form of modern slavery (In: SGEM Conference on Psychology & Psychiatry, Sociology & Healthcare, Education Conference Proceedings. Volume 2. (pp.131-137) Sofia: STEF92 Technology Ltd).
2. Allamby, L., Bell, J., Hamilton, J., Hansson, U., Jarman, N., Potter, M. and Toma, S. (2011). Forced labour in Northern Ireland. (York: Joseph Rowntree Foundation).
3. Anderson, B. and Rogaly, B. (2005). Forced labour and migration to the UK. (Oxford: COMPAS).
4. Andrees, B. (2008). Forced labour and trafficking in Europe: how people are trapped in, live through and come out. (Geneva: International Labour Organization).
5. Antal, I. and Laszlo, E. (2015). The Situation of Human Trafficking for Labour Exploitation in Romania. (In: SGEM Conference on Psychology & Psychiatry, Sociology & Healthcare, Education Conference Proceedings. Volume 1. (pp. 1031-1038) Sofia: STEF92 Technology Ltd).
6. Anti-Slavery International. (2014). Trafficking for forced criminal activities and begging in Europe: exploratory study and good practice examples. (London: Anti-Slavery International).
7. Aronowitz, A. (2009). “The smuggling - trafficking nexus and the myths surrounding human trafficking”. Sociology of Crime Law and Deviance, 13, 107-128.
8. Aronowitz, A., Theuermann, G. and Tyurykanova, E. (2010). Analysing the business model of trafficking. (Vienna: Organization for Security and Co-operation in Europe)
9. Balch, A. (2012). Regulation and enforcement to tackle forced labour in the UK. (York: Joseph Rowntree Foundation).
10. Beddoe, C. (2007). Missing Out: A Study of Child Trafficking in the North-West, North-East and West Midlands. (London: ECPAT UK).
11. Belser, P. (2005). Forced labour and human trafficking: estimating the profits. (Geneva: International Labour Organization).
12. Belser, P., de Cock, M. and Mehran, F. (2005). ILO Minimum estimates of forced labor in the world. (Geneva: International Labour Organization).
13. Bokhari, F. (2009). Stolen futures: trafficking for forced child marriage in the UK. (London: ECPAT UK).
14. Calandruccio, G. (2005). “A Review of Recent Research on Human Trafficking in the Middle East”. International Migration, 43(1-2), 267-299.
15. Child Exploitation and Online Protection Centre. (2011). Child Trafficking Update: October 2011. (London: Child Exploitation and Online Protection Centre).
16. Child Exploitation and Online Protection Centre. (2010). Strategic Threat Assessment Child Trafficking in the UK. (London: Child Exploitation and Online Protection Centre).
17. Child Exploitation and Online Protection Centre. (2010). The trafficking of children into and within the UK for benefit fraud purposes. (London: Child Exploitation and Online Protection Centre).
18. Child Exploitation and Online Protection Centre. (2009). Strategic Threat Assessment Child Trafficking in the UK. (London: Child Exploitation and Online Protection Centre).
19. Children's Society. (2010). Hidden children - separated children at risk. (London: Children’s Society).
20. Clark, N. (2013). Detecting and tackling forced labour in Europe. (York: Joseph Rowntree Foundation).
21. Craig, G., Gaus, A., Wilkinson, M., Skrivankova, K., and McQuade, A. (2007). Contemporary slavery in the UK: overview and key issues. (York: Joseph Rowntree Foundation).
22. De Angelis, M. I. (2012). Human trafficking: women’s stories of agency. (Hull: University of Hull).
23. De Jonge, B. (2005). Eurojust and human trafficking: the state of affairs. (The Hague: Eurojust).
24. Degirmencioglu, S.M., Acar, H. and Acar Y.B. (2008). “Extreme forms of child labour in Turkey”. Children and Society, 22(3), 191-200.
25. Delap, E. (2009). Begging for change: research findings and recommendations on forced child begging. (London: Anti-Slavery International).
26. Dettmeijer-Vermeulen, C., Boot-Matthijssen, M., van Dijk, E., de Jonge van Ellemeet, H. and Smit, M. (2008). Trafficking in Human Beings. Sixth report of the Dutch National Rapporteur. (The Hague: Office of the Dutch National Rapporteur on Trafficking in Human Beings).
27. Dettmeijer-Vermeulen, C., Boot-Matthijssen, M., van Dijk, E., de Jonge van Ellemeet, H., Koster, D. and Smit, M. (2006). Trafficking in Human Beings. Fifth report of the Dutch National Rapporteur. (The Hague: Office of the Dutch National Rapporteur on Trafficking in Human Beings).
28. Dottridge, M. (2006). Action to Prevent Child Trafficking in South Eastern Europe: A Preliminary Assessment. (Geneva: UNICEF and Terre des hommes).
29. Dowling, S., Moreton, K. and Wright, L. (2007). Trafficking for the purposes of labour exploitation: a literature review. (London: Home Office).
30. Equality and Human Rights Commission. (2011). Inquiry into Human Trafficking in Scotland: Report of the Equality and Human Rights Commission. (Glasgow: Equality and Human Rights Commission).
31. Esson, J. (2015). “Better Off at Home? Rethinking Responses to Trafficked West African Footballers in Europe”. Journal of Ethnic and Migration Studies, 41(3), 512-530.
32. European Roma Rights Centre and People in Need. (2011). Breaking the silence: Trafficking in Romani Communities. (Budapest: European Roma Rights Centre).
33. European Union Agency for Fundamental Rights (2015). Severe labour exploitation: workers moving within or into the European Union. (Vienna: European Union Agency for Fundamental Rights).
34. Europol. (2015). The THB Financial Business Model: Assessing the Current State of Knowledge. (The Hague: Europol).
35. Eurostat. (2015). Trafficking in human beings. 2015 edition. (Luxembourg: Publications Office of the European Union).
36. Eurostat. (2013). Trafficking in human beings. 2013 edition. (Luxembourg: Publications Office of the European Union).
37. Gavra, D. R., and Tudor, D. A. (2015). Addressing the Problem: Institutional Factors that Facilitate Human Trafficking and Potential Preventative Measures Through Communication (In: Redefining Community in Intercultural Context, RCIC’15. (pp. 205-2010). Brasov: Editura Acad Fortelor Aeriene Henri Coanda).
38. Geddes, A., Craig, G., Scott, S., Ackers, L., Robinson, O. and Scullion, D. (2013). Forced labour in the UK. (York: Joseph Rowntree Foundation).
39. Gjermeni, E., Van Hook, M.P., Gjipali, S., Xhillari, L., Lungu, F. and Hazizi, A. (2008). “Trafficking of children in Albania: Patterns of recruitment and reintegration”. Child Abuse and Neglect, 32(10), 941-948.
40. GRETA. (2012). Report concerning the implementation of the Council of Europe Convention on Action Against trafficking in human beings by the United Kingdom. (Strasbourg: GRETA).
41. Hales, L. and Gelsthorpe, L. (2011). “Research on criminalisation of migrant women”. Prison Service Journal, 198, 33-37.
42. Heil, E. and Nichols, A. (2015). Human Trafficking the Midwest: A Case Study of St. Louis and the Bi-State Area. (Beaverton: Ringgold Inc.).
43. Home Affairs Committee. (2009). The trade in human beings: human trafficking in the UK. Sixth report of Session 2008-09. Volume 1: report, together with formal minutes. (London: The Stationery Office).
44. Hunzinger, L. and Coffey, P. (2003). First Annual Report on Victims of Trafficking in South-Eastern Europe. (Geneva: International Organization for Migration).
45. Interdepartmental Ministerial Group on Human Trafficking. (2012). First annual report of the Inter-Departmental Ministerial Group on Human Trafficking*.* (London: The Stationery Office).
46. International Centre for Migration Policy Development. (2014). Current Anti-trafficking Response in Turkey and the Way Forward. (Vienna: International Centre for Migration Policy Development).
47. International Centre for Migration Policy Development. (2012). The Way Forward in Establishing Effective Transnational Referral Mechanisms: A Report Based on Experiences in Cases of Human Trafficking in South-Eastern Europe. (Vienna: International Centre for Migration Policy Development).
48. International Centre for Migration Policy Development. (2011). Transatlantic Journeys: An exploratory research on human trafficking from Brazil to Italy and Portugal. (Vienna: International Centre for Migration Policy Development).
49. International Centre for Migration Policy Development. (2010). Trafficking in Human Beings in Croatia: An Assessment Focusing on Labour Exploitation. (Vienna: International Centre for Migration Policy Development).
50. International Centre for Migration Policy Development. (2010). Study on the assessment of the extent of different types of Trafficking in Human Beings in EU countries. (Vienna: International Centre for Migration Policy Development).
51. International Centre for Migration Policy Development, and Department for Equal Opportunities - Presidency of the Council of Ministers, Italy. (2010). Study on Post-Trafficking Experiences in the Czech Republic, Hungary, Italy and Portugal. (Vienna: International Centre for Migration Policy Development).
52. International Centre for Migration Policy Development. (2007). Listening to Victims: Experiences of identification, return and assistance in South-Eastern Europe. (Vienna: International Centre for Migration Policy Development).
53. International Centre for Migration Policy Development, ECORYS, ECPAT Austria, Terre des hommes, Save the Children Denmark, Save the Children Italia, and Save the Children Romania. (2012). Report for the Study on Typology and Policy Responses to Child Begging in the EU. (Publication location not provided: European Commission).
54. International Centre for Migration Policy Development, and International Labour Organization. (2008). Labour Exploitation and Trafficking in Azerbaijan: An Exploratory Overview. (Vienna: International Centre for Migration Policy Development).
55. International Labour Office. (2009). Operational indicators of trafficking in human beings: results from a Delphi survey implemented by the ILO and the European Commission. (Geneva: International Labour Organization).
56. International Labour Office. (2009). The cost of coercion: Global report under the follow-up to the ILO Declaration on Fundamental Principles and Rights at Work. (Geneva: International Labour Organization).
57. International Labour Office. (2006). The end of child labour: Within reach. (Geneva: International Labour Organization).
58. International Organization for Migration. (2010). The Causes and Consequences of Re-trafficking: Evidence from the IOM Human Trafficking Database. (Geneva: International Organization for Migration).
59. International Organization for Migration. (2004). Changing patterns and trends of trafficking in persons in the Balkan region. (Geneva: International Organization for Migration).
60. International Organization for Migration and European Commission. (2002). Trafficking in Unaccompanied Minors in the European Union: Belgium, France, Greece, Italy, The Netherlands, Spain. (Geneva: International Organization for Migration).
61. Jarman, N. (2014). Forced labour in Northern Ireland: an update. (York: Joseph Rowntree Foundation).
62. Jokinen, A. and Ollus, N. (2013). Recruitment, Exploitation of Migrant Workers and Labour Trafficking in Finland, Sweden, Estonia and Lithuania: Introduction and Research Findings. (In N. Ollus, A. Jokinen and M. Joutsen (Eds.), Exploitation of migrant workers in Finland, Sweden, Estonia and Lithuania: uncovering the links between recruitment, irregular employment practices and labour trafficking, (pp. 9-28) Helsinki: HEUNI).
63. Jokinen, A. and Ollus, N. (2011). Trafficking for forced labour: Project Summary and Conclusions. (In A. Jokinen, N. Ollus and L. Aromaa (Eds.), Trafficking for forced labour and labour exploitation in Finland, Poland and Estonia (pp. 312-343) Helsinki: HEUNI).
64. Kangaspunta, K. (2004). “Mapping the inhuman trade: preliminary findings of the database in trafficking in human beings”. Crime and Society, 3(1), 81-103.
65. Kelly, E. (2009). Bordering on concern: child trafficking in Wales. (London: ECPAT UK).
66. Kelly, L. (2005). "'You can find anything you want': A critical reflection on research on trafficking in persons within and into Europe". International Migration, 43(1/2), 235-265
67. Kelly, L. (2005). Fertile fields: trafficking in persons in Central Asia. (Geneva: International Organization for Migration).
68. Laczko, F. and Gramegna, M. (2003). “Developing Better Indicators of Human Trafficking. Brown Journal of World Affairs”, 10(1), 179-191
69. Lalani, M. and Metcalf, H. (2012). Forced labour in the UK: the business angle. (York: Joseph Rowntree Foundation).
70. Lebov, K. (2010). “Human trafficking in Scotland”. European Journal of Criminology, 7(1), 77-93.
71. Leman, J. and Janssen, S. (2010). “Bulgarian Human Trafficking in Belgium and Proactive Learning Entrepreneurship: Developments 2002-2009”. Migracijske i etničke teme, 26(1), 7-26.
72. Lewis, H., Dwyer, P., Hodkinson, S. and Waite, L. (2014). Precarious lives: Forced labour, exploitation and asylum. (Bristol: Policy Press).
73. Lyneham, S. and Richards, K. (2014). Human Trafficking Involving Marriage and Partner Migration to Australia, AIC Reports Research and Public Policy Series 124 (Canberra: Australian Institute of Criminology).
74. Martynowicz, A., Toucas, S. and Caughey, A. (2009). The Nature and Extent of Human Trafficking in Northern Ireland: A scoping study. (Belfast: Equality Commmission for Northern Ireland).
75. Migrant Rights Centre Ireland. (2007). No Way Forward, No Going Back: Identifying the Problem of Trafficking for Forced Labour in Ireland. (Dublin: Migrant Rights Centre Ireland).
76. Musto, J. L. (2011). Institutionalizing Protection, Professionalizing Victim Management: Explorations of Multi-Professional Anti-Trafficking Efforts in the Netherlands and the United States. (Los Angeles: University of California).
77. National Crime Agency. (2015). National Referral Mechanism Statistics: End of Year Summary 2014. (No publication location given: National Crime Agency).
78. National Crime Agency. (2014). NCA strategic assessment: the nature and scale of human trafficking in 2013. (No publication location given: National Crime Agency).
79. National Crime Agency. (2014). National Referral Mechanism Statistics 2013. (No publication location given: National Crime Agency).
80. National Police Board. (2012). Trafficking in human beings for sexual and other purposes. Situation report 13. (Stockholm: National Police Board)
81. National Police Board. (2011). Trafficking in human beings for sexual and other purposes. Situation report 12. (Stockholm: National Police Board)
82. National Police Board. (2010). Trafficking in human beings for sexual and other purposes. Situation report 11. (Stockholm: National Police Board)
83. National Police Board. (2009). Trafficking in human beings for sexual and other purposes. Situation report 10. (Stockholm: National Police Board)
84. National Criminal Police. (2007). Trafficking in human beings for sexual and other purposes: Situation Report 9. January 1 - December 31 2006. (Stockholm: National Criminal Police).
85. National Rapporteur on Trafficking in Human Beings and Sexual Violence against Children. (2013). Trafficking in Human Beings. Ninth report of the Dutch National Rapporteur. (The Hague: Office of the Dutch National Rapporteur on Trafficking in Human Beings and Sexual Violence against Children).
86. National Rapporteur on Trafficking in Human Beings. (2010). Human Trafficking- ten years of independent monitoring. (The Hague: Dutch National Rapporteur on Trafficking in Human Beings).
87. National Rapporteur on Trafficking in Human Beings. (2009). Trafficking in Human Beings. Seventh report of the Dutch National Rapporteur. (The Hague: Dutch National Rapporteur on Trafficking in Human Beings).
88. Newton, P. J., Mulcahy, T. M., and Martin, S. E. (2008). Finding Victims of Human Trafficking. (Bethesda: University of Chicago).
89. Nordic Council of Ministers. (2013). Trafficking in human beings in working life. Report from a Nordic conference in Helsinki, Finland 27-28 November 2012. (Copenhagen: Norden).
90. Oram, S., Ostrovschi, N.V., Gorceag, V.I., Hotineanu, M.A., Gorceag, L., Trigub, C. and Abas, M.A. (2012). "Physical health symptoms reported by trafficked women receiving post-trafficking support in Moldova: Prevalence, severity and associated factors". BMC Women’s Health, 12(20), 1-9.
91. Oram, S., Stöckl, H., Busza, J., Howard, L. and Zimerman, C. (2012). “Prevalence and Risk of Violence and the Physical, Mental and Sexual Health Problems Associated with Human Trafficking: Systematic Review”, PLoS Medicine, 9(5).
92. Organised Crime Task Force. (2015). Annual Report and Threat Assessment 2015. (No publication location given: Organised Crime Task Force).
93. Organised Crime Task Force. (2014). 2014 Annual Report and Threat Assessment. (No publication location given: Organised Crime Task Force).
94. Organised Crime Task Force. (2013). Annual Report and Threat Assessment 2013. (No publication location given: Organised Crime Task Force).
95. Organised Crime Task Force. (2012). Annual Report and Threat Assessment 2012. (No publication location given: Organised Crime Task Force).
96. Organised Crime Task Force. (2011). Annual Report 2011. (No publication location given: Organised Crime Task Force).
97. Organised Crime Task Force. (2010). 2010 Annual Report and Threat Assessment. (No publication location given: Organised Crime Task Force).
98. Organization for Security and Co-operation in Europe. (2014). Ending exploitation, ensuring that businesses do not contribute to trafficking of human beings. Duties of states and the private sector. (Vienna: Organization for Security and Co-operation in Europe)
99. Organization for Security and Co-operation in Europe. (2008). Human trafficking for labour exploitation, forced and bonded labour: Identification, prevention, prosecution. (Vienna: Organization for Security and Co-operation in Europe).
100. Organization for Security and Co-operation in Europe. (2003). Trafficking in Human Beings in Southeastern Europe. (Vienna: Organization for Security and Co-operation in Europe).
101. Oude Breuil, B. (2008). “Precious Children in a Heartless World'? The Complexities of Child Trafficking in Marseille”. Children and Society, 22(3), 223-234.
102. Pearce, J. J., Hynes, P. and Bovarnick, S. (2009). Breaking the wall of silence: Practitioners' responses to trafficked children and young people. (London: NSPCC).
103. Petrunov, G. (2014). “Human Trafficking in Eastern Europe: The Case of Bulgaria”. Annals of the American Academy of Political and Social Science, 653(1), 162-182.
104. Potter, M., and Hamilton, J. (2014). “Picking on vulnerable migrants: Precarity and the mushroom industry in Northern Ireland”. Work, and Employment and Society, 28(3), 390-406.
105. Rijken, C. (2011). Challenges and Pitfalls in Combating Trafficking in Human Beings for Labour Exploitation. (In C. Rijken (Ed.) Combatting trafficking in human beings for labour exploitation (pp. 393-424) Nijmegen: Wolf Legal Publishers).
106. Ruwanpura, K. and Rai, P. (2004). Forced labour: definitions, indicators and measurement. (Geneva: International Labour Organization).
107. Ryazantsev, S.V, Karabulatova, I.S., Yureevna, S.S., Evgenyevna, P.E. and Vladimirovich, M.R. (2015). “Modern aspects of human trafficking in the context of labor exploitation and irregular labor migration in the Russian Federation”. Mediterranean Journal of Social Sciences, 6(3), 67-72.
108. Savona, E., Belli, R., Curtol, F., Decarli, S. and Di Nicola, A. (2003). Trafficking in persons and smuggling of migrants into Italy. (No publication location given: Transcrime).
109. Scott, S., Craig, G. and Geddes, A. (2012). Experiences of forced labour in the UK food industry. (York: Joseph Rowntree Foundation).
110. Scottish Parliament. (2010). Equal opportunities committee 5th report. Inquiry into migration and trafficking. (Edinburgh: Scottish Parliament).
111. Serious Organised Crime Agency. (2013). UKHTC Strategic Assessment on the Nature and Scale of Human Trafficking in 2012. (No publication location given: Serious Organised Crime Agency).
112. Serious Organised Crime Agency. (2013). National Referral Mechanism Statistics October to December 2012. (No publication location given: Serious Organised Crime Agency).
113. Serious Organised Crime Agency. (2013). National Referral Mechanism Statistics July to September 2012. (No publication location given: Serious Organised Crime Agency).
114. Serious Organised Crime Agency. (2012). UKHTC: a baseline assessment on the nature and scale of human trafficking in 2011. (No publication location given: Serious Organised Crime Agency).
115. Serious Organised Crime Agency. (2012). National Referral Mechanism Statistics April to June 2012. (No publication location given: Serious Organised Crime Agency).
116. Serious Organised Crime Agency. (2012). National Referral Mechanism Statistics January to March 2012. (No publication location given: Serious Organised Crime Agency).
117. Shelley, L. (2014). Human smuggling and trafficking into Europe: a comparative perspective. (Washington, D.C.: Migration Policy Institute).
118. Siegel, D. (2014). Mobile Banditry: East and Central European Itinerant Criminal Groups in the Netherlands. (The Hague: Eleven International Publishing).
119. Skrivankova, K. (2006). Trafficking for forced labour: a UK country report. (York: Joseph Rowntree Foundation).
120. Somerset, C. (2004). Cause for concern?: London social services and child trafficking. (London: ECPAT UK).
121. Surtees, R. (2007). Labour trafficking in South Eastern Europe: developing prevention and assistance programmes. (Vienna: Nexus Institute to Combat Human Trafficking).
122. Surtees, R. (2008). Trafficking of men: a trend less considered. (Geneva: International Organization for Migration).
123. Surtees, R. (2014). At sea: The trafficking of seafarers and fishers from Ukraine. (In M. Dragiewicz (Ed.), Global human trafficking: critical issues and contexts (pp.57-75) Abingdon: Routledge).
124. Tamas, A., Moise, A., Predut, C. and Medvichi, N. (2013). Trafficking in Persons for Begging- Romania Study. (No publication location given: Schweizerische Eidgenossenschaft and ANITP).
125. Terre des hommes. (2003). The trafficking of Albanian children in Greece. (Le Mont-sur-Lausanne: Terre des hommes).
126. The Anti-Trafficking Monitoring Group. (2012). All change: Preventing trafficking in the UK. (London: Anti-Slavery International).
127. The Anti-Trafficking Monitoring Group. (2010). Wrong kind of victim. (London: Anti-Slavery International).
128. The Slavery Working Group. (2013). It happens here: equipping the UK to fight modern slavery. (London: The Centre for Social Justice).
129. TNS OBOP and the British Embassy Warsaw. (2010). Public awareness of risks involved in human trafficking and taking up work abroad. (No publication location given: TNS OBOP).
130. Turner-Moss, E., Zimmerman, C., Howard, L.M. and Oram, S. (2013). “Labour Exploitation and Health: A Case Series of Men and Women Seeking Post-Trafficking Services”. Journal of Immigrant and Minority Health, 16, 473-480.
131. Tyldum, G. (2013). “Dependence and human trafficking in the context of transnational marriage”. International Migration, 51(4),103-115.
132. UNICEF and Save the Children Norway. (2002). Research on Child Trafficking in Bosnia and Herzegovina. (No publication location provided: UNICEF).
133. United Nations Office on Drugs and Crime. (2012). Global Report on Trafficking in Persons. (Vienna: United Nations Office on Drugs and Crime).
134. United Nations Office on Drugs and Crime. (2014). Global Report on Trafficking in Persons. (Vienna: United Nations Office on Drugs and Crime).
135. United Nations Office on Drugs and Crime. (2009). Trafficking in persons: a global report. (Vienna: United Nations Office on Drugs and Crime).
136. United Nations Office on Drugs and Crime. (2006). Trafficking in persons: global patterns. (Vienna: United Nations Office on Drugs and Crime).
137. U.S. Department of State. (2014). Trafficking in Persons Report June 2014. (Washington, D.C.: U.S. Department of State).
138. U.S. Department of State. (2013). Trafficking in Persons Report June 2013. (Washington, D.C.: U.S. Department of State).
139. U.S. Department of State. (2012). Trafficking in Persons Report June 2012. (Washington, D.C.: U.S. Department of State).
140. U.S. Department of State. (2011). Trafficking in Persons Report June 2011. (Washington, D.C.: U.S. Department of State).
141. U.S. Department of State. (2010). Trafficking in Persons Report Tenth Edition June 2010. (Washington, D.C.: U.S. Department of State).
142. U.S. Department of State. (2009). Trafficking in Persons Report June 2009. (Washington, D.C.: U.S. Department of State).
143. U.S. Department of State. (2008). Trafficking in Persons Report June 2008. (Washington, D.C.: U.S. Department of State).
144. U.S. Department of State. (2007). Trafficking in Persons Report June 2007. (Washington, D.C.: U.S. Department of State).
145. U.S. Department of State. (2006). Trafficking in Persons Report June 2006. (Washington, D.C.: U.S. Department of State).
146. U.S. Department of State. (2005). Trafficking in Persons Report June 2005. (Washington, D.C.: U.S. Department of State).
147. U.S. Department of State. (2004). Trafficking in Persons Report June 2004. (Washington, D.C.: U.S. Department of State).
148. U.S. Department of State. (2003). Trafficking in Persons Report June 2003. (Washington, D.C.: U.S. Department of State).
149. U.S. Department of State. (2002). Trafficking in Persons Report June 2002. (Washington, D.C.: U.S. Department of State).
150. U.S. Department of State. (2001). Trafficking in Persons Report July 2001. (Washington, D.C.: U.S. Department of State).
151. Villacampa, C. and Torres, N. (2014). “Trafficked Women in Prison: The Problem of Double Victimisation”. European Journal of Criminal Policy Research, 21, 99-115.
152. Wilkinson, M., Craig, G. and Gaus, A. (2010). Forced labour in the UK and the GLA. (Hull: WISE).

**Appendix 7: Full breakdown of the quality assessment scores**

| **Assessment criteria^1^** | **Publication** | | | | | | | |
| --- | --- | --- | --- | --- | --- | --- | --- | --- |
|  | Antal & Laszlo, 2015 | Gavra & Tudor, 2015 | Jokinen & Ollus, 2011 | Oram et al., 2012b | Rijken, 2011 | Tamas *et al*., 2013 | Turner-Moss *et al*., 2014 | UNICEF & Save the Children Norway, 2012 |
| Does the study have a clear, explicit and feasible research question(s) or statement of intent(s) | 0 | 1 | 1 | 2 | 2 | 1 | 2 | 1 |
| Is the overall study design clear and appropriate? | 1 | 0 | 1 | 2 | 1 | 1 | 2 | 1 |
| Have ethical considerations been explicitly discussed and properly managed? | 0 | 0 | 2 | 2 | 0 | 1 | 2 | 0 |
| Is the sampling method explicit and appropriate? | 1 | 0 | 1 | 2 | 0 | 1 | 2 | 0 |
| Is the sample itself adequate? | 1 | 0 | 1 | 1 | 0 | 1 | 1 | 0 |
| Is the analytical procedure transparent and appropriate? | 0 | 0 | 1 | 2 | 0 | 0 | 2 | 0 |
| Are the results clear and precise? | 1 | 1 | 1 | 2 | 1 | 0 | 1 | 0 |
| Are limitations identified and accounted for? | 0 | 0 | 0 | 2 | 0 | 1 | 2 | 1 |
| Are the conclusions properly grounded in the results? | 1 | 0 | 1 | 2 | 1 | 0 | 2 | 0 |
| **Total score (out of a maximum of 18)** | **5** | **2** | **9** | **17** | **5** | **6** | **16** | **3** |

*^1^To recap, each criterion was scored as follows: 0= Publication does not meet the criterion and/or does not contain sufficient information to assess whether it meets the criterion at all; 1= Publication partially meets the criterion and/or only contains information permitting a partial assessment of whether it meets the criteria; 2= Publication fully meets the criterion.*
